# Cycles of goal silencing and reactivation underlie complex problem-solving in primate frontal and parietal cortex

**DOI:** 10.1038/s41467-023-40676-1

**Published:** 2023-08-19

**Authors:** Kei Watanabe, Mikiko Kadohisa, Makoto Kusunoki, Mark J. Buckley, John Duncan

**Affiliations:** 1 Department of Experimental Psychology, University of Oxford, OX1 3SR Oxford, UK; 2 Graduate School of Frontier Biosciences, Osaka University, Osaka, 565-0871 Japan; 3 Graduate School of Medicine, Osaka University, Osaka, 565-0871 Japan; 4 MRC Cognition and Brain Sciences Unit, University of Cambridge, CB2 7EF Cambridge, UK

**Keywords:** Cognitive control, Working memory

## Abstract

While classic views proposed that working memory (WM) is mediated by sustained firing, recent evidence suggests a contribution of activity-silent states. Within WM, human neuroimaging studies suggest a switch between attentional foreground and background, with only the foregrounded item represented in active neural firing. To address this process at the cellular level, we recorded prefrontal (PFC) and posterior parietal (PPC) neurons in a complex problem-solving task, with monkeys searching for one or two target locations in a first cycle of trials, and retaining them for memory-guided revisits on subsequent cycles. When target locations were discovered, neither frontal nor parietal neurons showed sustained goal-location codes continuing into subsequent trials and cycles. Instead there were sequences of timely goal silencing and reactivation, and following reactivation, sustained states until behavioral response. With two target locations, goal representations in both regions showed evidence of transitions between foreground and background, but the PFC representation was more complete, extending beyond the current trial to include both past and future selections. In the absence of unbroken sustained codes, different neuronal states interact to support maintenance and retrieval of WM representations across successive trials.

## Abstract (web summary)

Simple working memory tasks show sustained neural firing in frontal and parietal cortex. Here, the authors show cycles of target silencing and reactivation that are more restricted to single targets in parietal than frontal cortex.

## Introduction

It has been widely held that information maintenance in working memory (WM) is accomplished by sustained firing of so-called “delay neurons” in the frontoparietal network, and the disappearance of sustained activity is regarded as loss of memory^1–4^. Recently, evidence has been accumulating which suggests the existence of WM maintenance independent of sustained neuronal firing^5,6^. This view proposes that WM items are associated with different levels of neural activation, depending on the degree of relevance to immediate behavior. Neural representation for a WM item which is highly relevant in the current behavioral context is attentionally foregrounded and elicits increased firing of related neurons. By contrast, neural representations for less relevant items are temporarily silent or much diminished; these representations, however, are still maintained in memory latently, and can be reactivated once they return to the focus of attention^5,7–9^. This neural phenomenon is often referred to as activity-silent maintenance of working memory^6^, though it is hard in principle to distinguish truly silent codes, based, for example, on short-term synaptic change^10^, from codes so diminished as to escape detection.

To study these different modes of WM, it is essential to use behavioral situations in which a subject attentively foregrounds or backgrounds relevant memory items as needed. However, available cellular-level evidence on WM depends almost solely on delay paradigms which have very simple memory requirements completed within a single trial (e.g., an oculomotor delayed-response task^2,11^ and an object delayed match-to-sample task^12^). In a typical delay task, subjects retain an externally presented sensory stimulus (cue) for a few seconds before making a response indicated by that cue (though see refs. ^13^,^14^ for examples of more complex, multiple-item delay tasks). Task processing is usually completed within a single trial and does not share context with preceding or following trials. Thus, these delay paradigms are not well-suited to examine a memory representation switching back and forth between attentionally foregrounded (active) and backgrounded (silent) states. Instead, these neural phenomena would be better captured by a multistep task that involves multiple memory items and sequential action planning.

In human studies, there is ample evidence that the frontoparietal cortex is involved in complex, sequential action planning (e.g., refs. ^15–17^). In formal neuropsychological tests, such as the Multiple Errands Test and the Six Elements Test, participants are required to efficiently complete a number of subtasks within a time limit, while they are free to decide in what order they solved each subtask^16,18^. Frontal patients have shown marked deficits in executing action plans in an orderly fashion. It seems that without PFC, it is difficult to sequentially execute the correct action at the correct time to accomplish an overall objective. Similarly, frontal patients commonly show “goal neglect”, or neglect of a part of a task’s rules even though these rules are understood^19^; the rate of goal neglect increases with task complexity, and in complex settings, similar behavior appears in members of the normal population^20^. To date, there are few neurophysiology studies using complex, sequential goal-directed tasks (for an early example, see ref. ^21^), and much remains unknown regarding the specific involvement of frontal and parietal cortices.

In this study, we recorded neuronal activity across frontoparietal cortices in monkeys while they performed a sequential, spatial problem-solving task. In this task, monkeys were first required to search through a stimulus display and find one or two currently rewarded locations. In subsequent trials, monkeys used this knowledge to guide re-selection of the same locations. Our results address the distinct roles of frontal and parietal cortex in structuring complex, multistep behavior, including their roles in silencing and reactivation of target memory, and transitions from attentional foreground to background. We also show how the neuronal processes in this complex setting are related to the well-documented persistent state of neuronal firing in a classical delayed-response task.

## Results

**Fig. 1:**
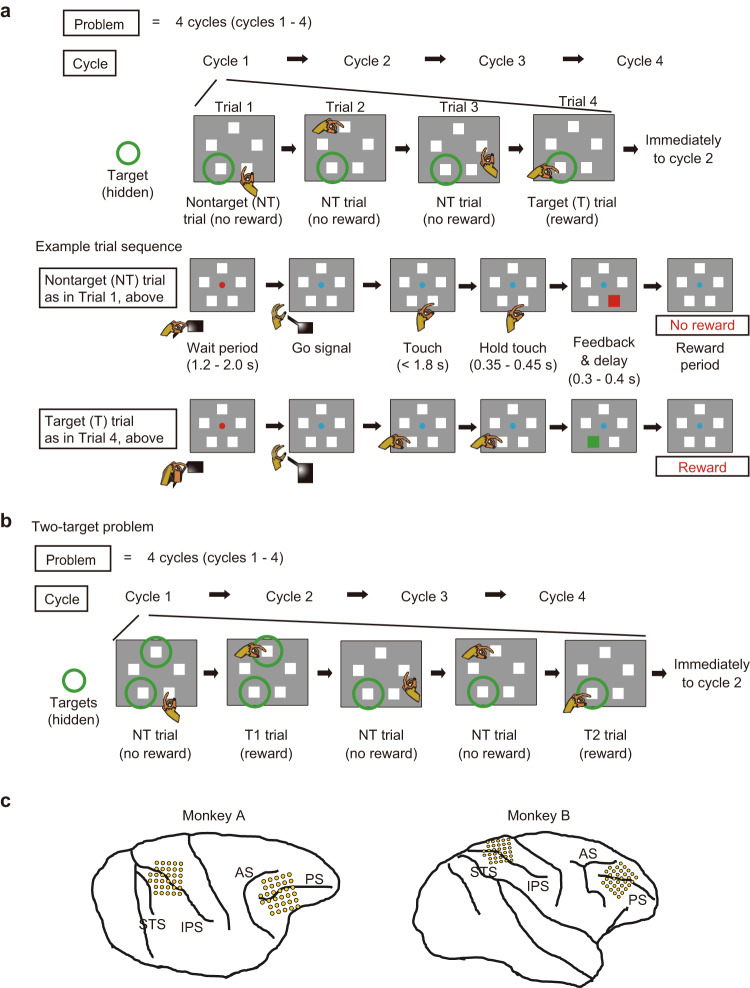
Behavioral task and recording locations. **a** Illustration of a one-target problem. In each problem, there was only a single target location to be discovered (green circle, not visible on actual display). Touching a target gave reward (T trial), whereas touching a nontarget gave no reward (NT trial). Each cycle was terminated immediately after a sole target location was selected (i.e., T trial). After the T location had been discovered in cycle 1, rewards in cycles 2 to 4 were given for selecting this target again. Optimally, therefore, cycles 2 to 4 each consisted of just a single trial. **b** Illustration of two-target problem. In each problem, two locations were targets (green circles, not visible on actual display). Each cycle was terminated after both targets were selected once (i.e., after T2 trial). **c** Recording locations for the two animals. Note that, to increase cell capture in animal A, the frontal array was repositioned (rotated) once within the chamber midway through the experiment; the figure shows electrode locations before this rotation. AS arcuate sulcus, IPS intraparietal sulcus, PS principal sulcus, STS superior temporal sulcus.

We obtained data from two monkeys (monkeys A and B) extensively trained in a complex, sequential spatial memory task. In each session, the monkeys worked through a series of problems, each consisting of four cycles of trials (Fig. 1a, top). In each trial (Fig. 1a, bottom, “Example trial sequence”), the monkey was presented with an array of five small placeholders (square or circle) which marked five locations surrounding a central fixation point (FP). Shapes of placeholders were alternated between problems, and this change in shape (along with additional cues, see “Methods”) served to indicate the start of a new problem. After a variable interval (1.2–2 s), on receipt of a go signal (Go, change of FP color), the monkey reached out to touch one location. In each problem, one (one-target problems, Fig. 1a) or two (two-target problems, Fig. 1b) locations were defined as targets. Touching a target location (thereafter termed target (T) trial in one-target problems, and T1 and T2 trials in two-target problems, Fig. 1b) brought a positive visual feedback and reward, while touching any other location (nontarget) brought negative feedback and no reward (thereafter termed nontarget (NT) trial, Fig. 1a, b). Note that in two-target problems, the labels, T1 and T2, indicate the order of target selection for each cycle, without taking into consideration both the order of initial target discovery in cycle 1 and the order of target selection in other cycles. Thus, cycle 1 of each problem was a search phase in which the monkey had to visit different locations on successive trials until the target or targets were discovered. In subsequent cycles, the optimal performance was to re-select the same target locations and avoid nontarget locations based on memory (memory-guided phase).

Trials continued in each cycle until all targets had been selected once. Thus in one-target problems, each cycle was terminated immediately after a T trial in which the monkey selected the sole target location. In two-target problems, the monkey was free to visit the two targets in each cycle in either order. For each target location, reward was only available the first time it was touched within each cycle; revisits brought negative feedback and no reward. Thus, after target discovery in cycle 1, optimal performance in subsequent cycles consisted of just a single trial for a one-target problem, and of two trials for a two-target problem. We blocked one-target and two-target problems in each recording session, so that monkeys knew the current number of targets. Additional cues reinforced the monkey’s knowledge of when each cycle and each problem were completed (see “Methods”). Across two monkeys, an average of 71 and 81 problems were performed for one-target and two-target problems, respectively, per session.

**Fig. 2:**
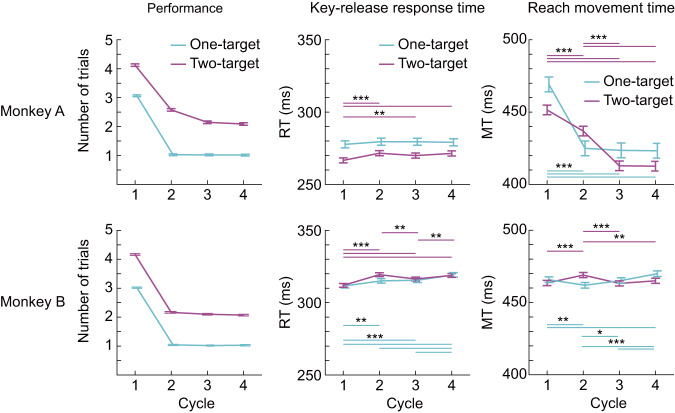
Behavioral results. Left: mean number of trials (location touches) per cycle. Middle: mean reaction time (RT) to release the start key after go signal. Right: mean movement time (MT) from key release to screen touch. In each panel, *n* = 44 and 40 sessions for monkeys A and B, respectively. Error bars indicate within-subject 95% confidence interval of the mean calculated by Loftus-Masson’s method. *P < 0.05, **P < 0.01, ***P < 0.001 from post hoc comparisons (Ryan’s procedure) following one-way repeated-measures ANOVA. Source data are provided as a Source Data file.

Behavioral data indicated that performance on one-target and two-target problems was near optimal across cycles 2–4 (Fig. 2). Key-release response time (RT) was significantly longer in later cycles. Time from key release to touch (reach movement time, MT) was significantly shorter in later cycles, though this was only seen in monkey A. These trends in the RT and MT data are consistent with more careful planning before movement in later cycles. Another notable feature is that both monkeys performed slightly worse in two-target problems than in one-target problems in cycles 2–4; while in two-target problems, both monkeys achieved ceiling (optimal) performance only by cycle 4, the performance in one-target problems was near optimal from cycle 2. The observed performance decrement in two-target problems can be naturally interpreted as evidence of capacity limitation in working memory. The complexity of the two-target problems, however, involved more than a requirement to remember two targets, with additional constraints on selection order (freedom to begin each cycle with either target, but then to avoid re-selection until the next cycle).

Neural recordings were made in the frontal and parietal cortex (Fig. 1c, see “Methods”). In PFC, recordings were made on dorsal and ventral prefrontal convexities, and within the principal sulcus. In PPC, recordings were made on the surface of the superior and inferior parietal lobules, and within the intraparietal sulcus. Prefrontal data were separated into dorsal and ventral regions, divided at the fundus of the principal sulcus. Parietal data were separated into superior (MIP/area 5) and inferior (LFP/area 7) regions, divided at the fundus of the intraparietal sulcus. In total, for both one-target and two-target problems, we recorded the activities of 498 neurons in the PFC (303 dorsal, 262/41, respectively, from monkeys A/B; 195 ventral, 149/46, respectively, A/B) and of 569 neurons in PPC (252 inferior, 177/75 from A/B; 317 superior, 245/72 from A/B).

**Fig. 3:**
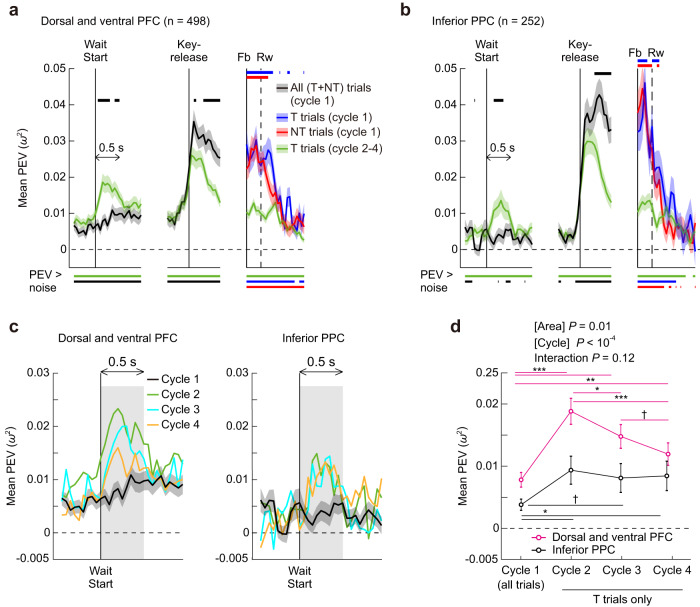
Location selectivity in one-target problems. **a** Time series of mean PEV (*ω*^2^) for location over all recorded cells in the dorsal and ventral PFC (*n* = 498). Shaded areas indicate SEM. Neural activities are aligned at the onsets of wait period (wait start), key release and visual feedback (Fb). Rw marks average time of reward. Black curve represents all trials (T and NT trials collapsed) in cycle 1. Blue and red represent T and NT trials in cycle 1, respectively, and green, T trials in cycles 2 to 4. Lower horizontal bars indicate periods of significant location selectivity for each trial type (PEV > noise, FDR-controlled permutation test, *P* < 0.05). Upper horizontal bars show significant difference between different trial types. Black: all trials (cycle 1) vs. T trials (cycle 2–4). Blue: T trials (cycle 1) vs T trials (cycle 2–4). Red: NT trials (cycle 1) vs T trials (cycle 2–4). **b** Same as in (**a**), but for the inferior PPC (*n* = 252). **c** Time series of PEV before and after the wait-start time for each individual cycle in PFC and PPC (*n* = 498 and 252, respectively). **d** Comparison of mean PEV in the wait period (shaded area in **c**) across cycles in PFC and PPC (*n* = 498 and 252, respectively). Error bars indicate SEM. ^†^*P* < 0.1, *P < 0.05, **P < 0.01, ***P < 0.001 in the post hoc multiple comparisons. Source data are provided as a Source Data file.

**Fig. 4:**
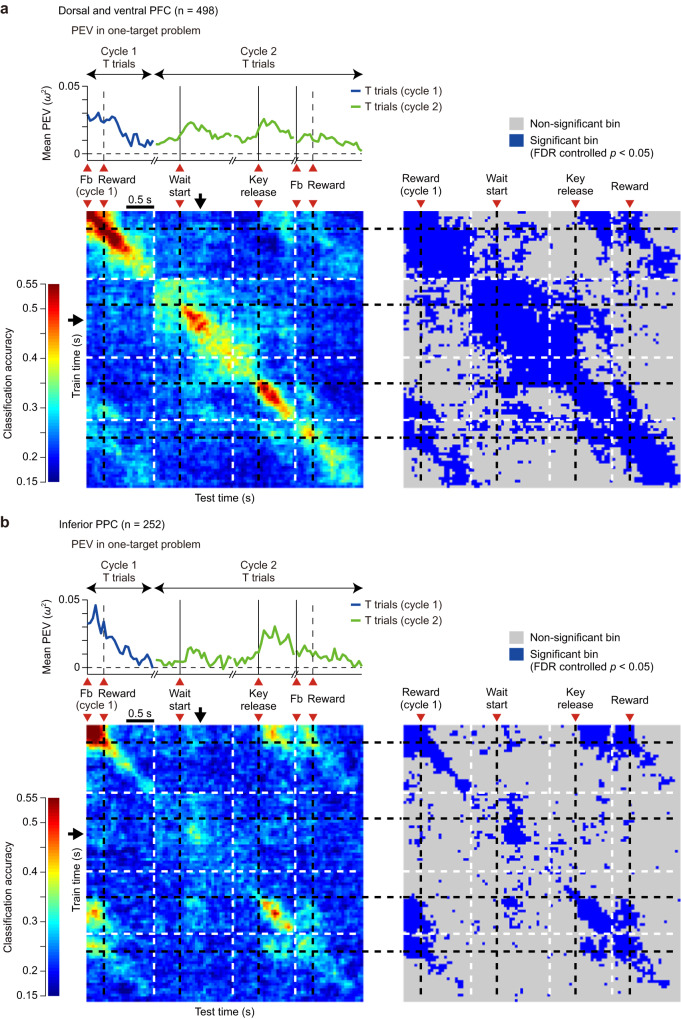
Similarity of location coding across different task periods and cycles in one-target problems. **a** Dorsal and ventral PFC. Results of cross-temporal decoding analysis in cycles 1 and 2 for all recorded cells (*n* = 498). Upper part: Time series of mean PEV, as in Fig. 3. Lower part: Cross-temporal decoding results. Time bins along both the abscissa and the ordinate correspond to analysis time points of the PEV time series above. The panel to the right illustrates time bins exhibiting significant classification accuracy which was calculated as the tail probability of the actual classification accuracy under the null distribution generated by running the decoding analysis with the location labels randomly shuffled. (FDR-controlled *P* < 0.05). Note that because the time window of this analysis did not include the wait period of cycle 1, the first trial of cycle 1 was not removed from this analysis. **b** Same as in (**a**), but for the inferior PPC (*n* = 252). Source data are provided as a Source Data file.

**Fig. 5:**
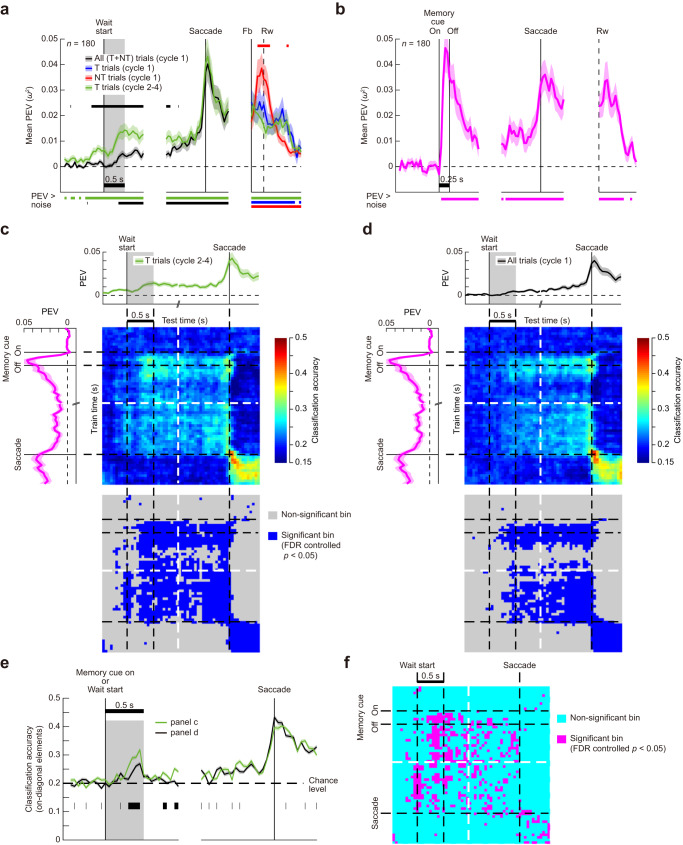
Relations between neural activity in the oculomotor sequential memory task and the MGS task. **a** Time series of population-averaged PEV in the oculomotor sequential memory task over all recorded cells (*n* = 180). Upper horizontal bars show significant difference between different trial types. Black: all trials (cycle 1) vs. T trials (cycles 2–4). Red: NT trials (cycle 1) vs T trials (cycles 2–4). Other conventions as in Fig. 3. **b** Time series of PEV in MGS over the same neural population as in (**a**) (*n* = 180). **c** Cross-temporal decoding analysis between T trials in cycles 2–4 of the sequential memory task and correct trials in MGS (*n* = 180). In the color-coded panel, time bins along the abscissa correspond to analysis time points of the PEV time series in the sequential memory task, as indicated by the PEV plot above the panel. Likewise, time bins along the ordinate correspond to time points of PEV in MGS. Other conventions as in Fig. 4. **d** Same as in (**c**), but for the comparison between all trials in cycle 1 and MGS. **e** Comparison of the on-diagonal classification accuracies between (**c**) and (**d**). Shaded areas indicate SEM which was calculated by using all values obtained in the resampling runs (see “Methods”). Lower horizontal bars indicate time bins with significant difference (FDR-controlled *P* < 0.05). **f** Time bins of significant difference in classification accuracies between (**c**) and (**d**). A time bin was deemed significant by meeting two conditions; (1) the bin showed significant difference in classification accuracies between (**c**) and (**d**); (2) the bin showed significantly above-chance classification in either panel (both FDR-controlled *P* < 0.05). Source data are provided as a Source Data file.

**Fig. 6:**
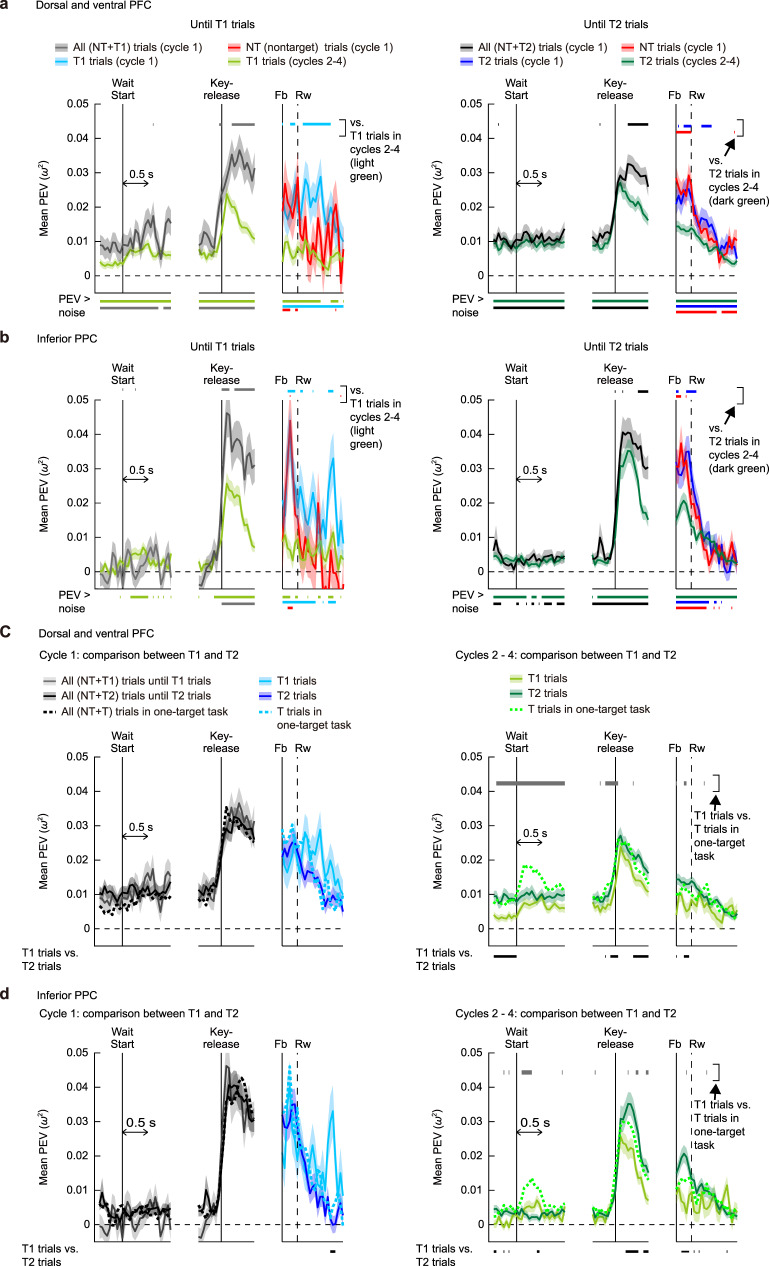
Location selectivity in two-target problems. **a** Time series of population-averaged PEV in the dorsal and ventral PFC (*n* = 498). Left: PEV for trials up to and including the T1 trials (“until T1”) in three trial types in cycle 1 (all trials, gray; T1 trials, cyan; NT trials, red) and T1 trials in cycles 2–4 (light green). Time periods of significant difference between each of the three trial types in cycle 1 versus T1 trials in cycles 2–4 are indicated by upper horizontal bars (FDR-controlled *P* < 0.05), the color of which indicates a trial type of cycle 1 data. Lower horizontal bars indicate periods of significant location selectivity (PEV > noise, permutation test, FDR-controlled *P* < 0.05). Right: same as in the left panel, but for the “until T2” trials in PFC. **b** Same as in (**a**), but for the inferior PPC (*n* = 252). **c** Comparison of PEV between T1 and T2 trials in the dorsal and ventral PFC, separately shown for cycle 1 (left) and cycles 2–4 (right). In cycle 1, all “until T1” and all “until T2” trials are included for wait and key-release periods. For reference, the PEV time series in the one-target case (Fig. 3a) is shown as dashed lines. Lower black horizontal bars indicate periods of significant difference in PEV between T1 and T2 trials. Before Fb in cycle 1, the comparison was made between all trials until T1 trial and those until T2 trial. Upper gray horizontal bars in the right panel indicate periods of significant difference in PEV between T1 trials in two-target problems and T trials in one-target problems. **d** Same as in (**c**), but for the inferior PPC. Shaded areas indicate SEM.

**Fig. 7:**
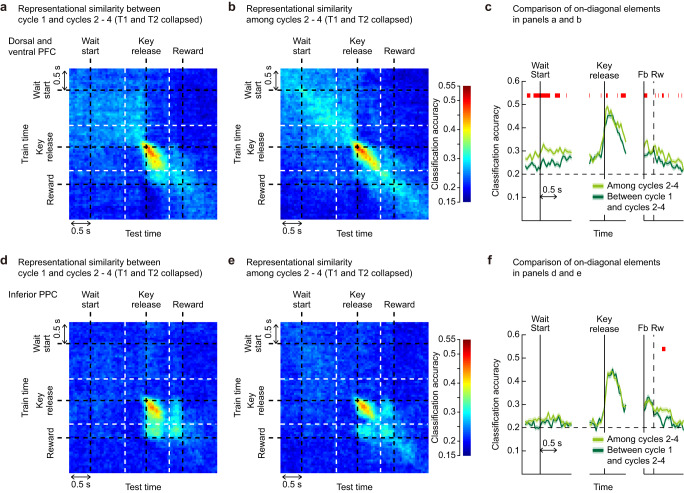
Evidence for processing of target memory during the wait period in two-target problems. **a** Between-phase analysis: similarity of location coding between cycle 1 (search phase) and cycles 2–4 (memory-guided phase) the dorsal and ventral PFC (*n* = 498). Classifier was first trained using all trials in cycle 1 (T1, T2, and NT trials), and then tested on all correct trials in cycles 2–4 (T1 and T2 trials). **b** Within-phase analysis: same as in (**a**), except that both training and test trials come from correct trials in cycles 2–4. **c** Comparison of classification accuracy between (**a**) and (**b**). Dark green: on-diagonal elements in (**a**) (similarity of location coding between cycle 1 and cycles 2–4). Light green: on-diagonal elements in (**b**) (similarity among cycles 2–4). Shaded areas indicate SEM which was calculated by using values obtained in the resampling runs (see “Methods”). Upper red horizontal bars indicate periods of significant difference (permutation test, FDR-controlled *P* < 0.05). **d**–**f** Same as in (**a**–**c**), but for the inferior PPC (*n* = 252). Source data are provided as a Source Data file.

**Fig. 8:**
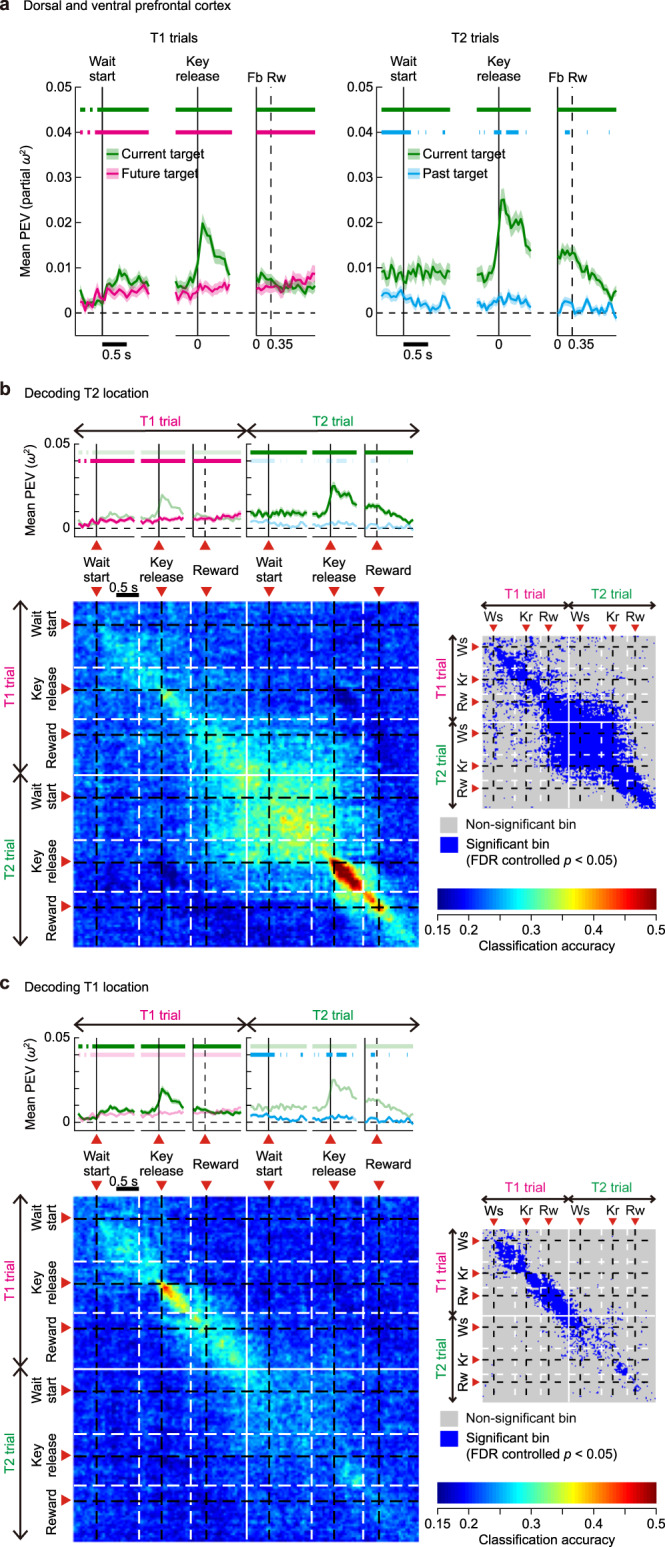
Encoding of past, present and future targets in perfect cycles of two-target problems in PFC. **a** Time series of PEV for current (green), future (magenta), and past (cyan) targets in T1 (left column) and T2 (right column) trials in the dorsal and ventral PFC (*n* = 498). Upper horizontal bars indicate periods of significant location selectivity (PEV > noise, FDR-controlled permutation test, *P* < 0.05) for current (green), future (magenta), and past (cyan) targets. **b** Evolution of T2 target representation across T1 and T2 trials in perfect cycles. Note that on-diagonal elements in upper-left quadrant and lower-right quadrant correspond to analysis time points of the PEV time series during T1 (magenta) and T2 trials (green), respectively, as indicated by the PEV plots above the panel. The right inset indicates time bins with significant classification accuracy (FDR-controlled *P* < 0.05). Ws wait start, Kr key release. **c** Same as in (**b**), but for changes in T1 target representation across T1 and T2 trials. Source data are provided as a Source Data file.

**Fig. 9:**
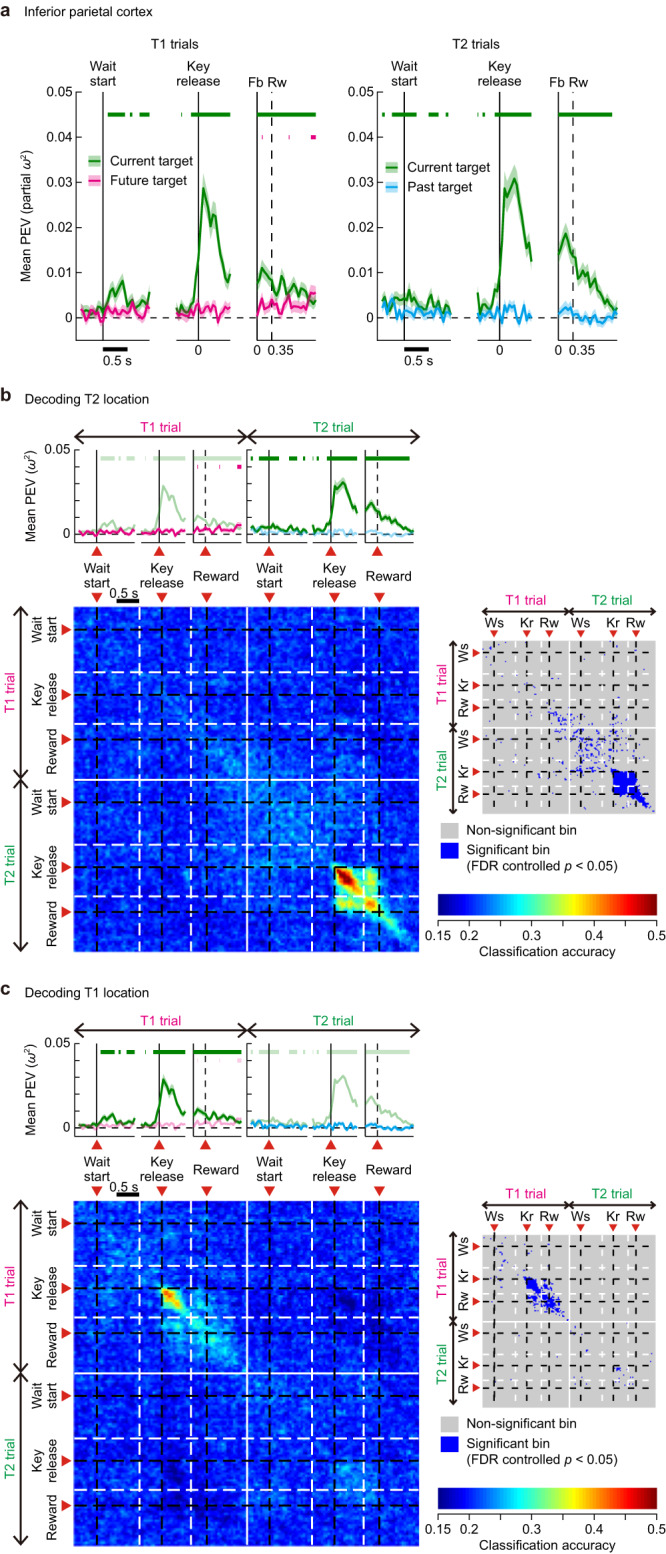
Encoding of past, present and future targets in perfect cycles of two-target problems in inferior PPC. **a**–**c** Same as in Fig. 8a–c, but for the inferior PPC (*n* = 252). Source data are provided as a Source Data file.

In the following sections, we first establish basic characteristics of location coding using results in one-target problems. Then, we consider how location coding is altered by the increased complexity and load of two-target problems. Note that, in each cycle, placeholders appeared at wait start of the first trial, then remained on the screen until the cycle ended. In cycle 1, any visual response to placeholder onset would have been confounded with the animal’s location choice, as each animal was strongly biased to begin his search with the same favored location. To remove this confound, the first trial of cycle 1 was removed from all analyses concerning the wait period of cycle 1. Specifically, for the one-target problems, this removal was applied in the analysis of Fig. 3, but not for Figs. 4 and 5. For the two-target problems, this removal was done in the analysis of Figs. 6 and 7, but not for Figs. 8 and 9. In the supplementary figures, this removal was done in Supplementary Figs. S1, S2, S6, S7, and S9. No similar issue applied to later cycles, as behavior was largely determined not by response bias but by the animal’s knowledge of the correct choice (see Fig. 2).

### Frontoparietal activity separates learning and retrieval

Using data from one-target problems, our initial neural analysis focused on comparison between cycle 1 (search) and cycles 2–4 (memory-guided). In cycle 1, the monkeys did not know which location was the target. As they searched through an array of placeholders, they were instructed by the feedback (Fb) signal as to whether the selected location was the target (T trial), to be revisited in subsequent cycles, or a nontarget (NT trial) that was to be avoided. In contrast, in cycles 2–4, as indicated by the near-perfect performance, the monkeys knew the target location and exploited this knowledge. Therefore, comparison of strength of selectivity for target location between cycle 1 and cycles 2–4 would reveal how the target memory is registered, retained and later used to guide behavior.

To provide an initial view, we calculated a time series of strength of location selectivity using an omega-squared PEV (proportion of explained variance, derived from one-way ANOVA with a factor of selected location on individual neurons and then averaged over neurons) (Eq. (1), “Methods”). We compared profiles of PEV between cycle 1 and cycles 2–4 averaged across the whole population of recorded cells in dorsal and ventral PFC combined (Fig. 3a) and inferior PPC (Fig. 3b). We collapsed the data from dorsal and ventral prefrontal cells because the response profiles of these two populations were highly similar (Supplementary Fig. S1a, b). We present the results from superior PPC only in the supplementary figures because the activity of this area showed little indication of critical involvement in task processing (Supplementary Fig. S1d). In cycle 1, because the monkey did not know the trial outcome prior to Fb, we collapsed T and NT trials (black curves, termed “all trials”). After Fb, we calculated PEV separately for T and NT trials (blue and red curves, respectively). For cycles 2–4, we calculated PEV only for T trials (green curves) due to the near-perfect performance of the monkeys (i.e., few NT trials).

First, we consider activity in the wait period (Fig. 1a, bottom), when the monkey maintained fixation, held the start key, and prepared to reach to the chosen screen location. Though behavior was closely matched in cycle 1 (search) and cycles 2–4 (retrieval), neural data were quite different. In dorsal and ventral PFC, as well as inferior PPC, retrieval of a known target location in cycles 2–4 was associated with a striking peak of location coding, arising shortly after onset of the wait start and declining by around 500 ms (Fig. 3a, b). In contrast, there was little hint of this specific involvement in retrieval for cells in superior PPC (Supplementary Fig. S1d).

For a further analysis of PEV around the wait-start time, we compared PEV in individual cycles. As shown in Fig. 3c, in PFC, there is a noticeable difference in the magnitude of PEV even among cycles 2–4; cycle 2 showed the largest peak in the PEV time series, and after that there was a monotonic decrease toward cycle 4. To quantify this effect, we performed a 2-by-4 two-way mixed-design ANOVA with factors area and cycle, on the mean PEV in the wait period of each recorded cell (0–500 ms from wait-start, gray shaded areas in Fig. 3c). As shown in Fig. 3d, the result showed that PEV in the wait period differed significantly depending on area and cycle (main effect of area, *F*_1,__748_ = 6.33, *P* = 0.01; cycle, *F*_3,__2244_ = 12.7, *P* < 10^−4^; interaction, *F*_3,__2244_ = 1.94, *P* = 0.12). Although the interaction was not significant, post hoc simple effect analyses confirmed that the aforementioned trend was highly significant in prefrontal cells (simple effect of cycle, *F*_3,__2244_ = 11.3, *P* < 10^−4^), with inferior parietal cells showing a similar but more moderate trend (*F*_3,__2244_ = 3.32, *P* = 0.02). In superior parietal cells, the effect of cycle did not reach significance (*P* = 0.5, Supplementary Fig. S1e, f). The results suggest a decreasing reliance on PFC with increasing experience of selecting the same, repeated target.

Second, we consider activity towards the end of each trial, as the animal prepared for and processed feedback. A variety of processes could contribute to location coding at this stage of the trial. At this time, the monkey’s touch was maintained at the selected screen location, and though eye position was not controlled, most commonly the animal fixated the selected location. In cycle 1, furthermore, feedback provided an important signal to remember the selected location either for revisit on later cycles, or to be avoided for the remainder of the problem. Corresponding to this requirement for location learning, the data showed substantially stronger location coding in cycle 1 as compared to cycles 2–4. Again, this result was striking in PFC, as well as in inferior PPC, but weak or absent in superior PPC (Supplementary Fig. S1**)**.

### Choice and feedback are associated with independent patterns of neural activity

Our next question was whether there are relations between Fb–Rw activity in cycle 1 and the wait-period activity in subsequent cycles. In all recording regions, and across cycles, target encoding on each trial waned soon after Rw time (Fig. 3a, b and Supplementary Fig. S1a–d). This suggests that during an ITI between cycles, target information was not strongly maintained in a sustained fashion from one cycle to another. As noted above, several factors could contribute to location signals in the Fb–Rw period, but strong coding in cycle 1 suggests a role in learning the target location. We wondered whether, when location signals reappeared at the start of each trial in cycles 2–4, they resembled those seen during cycle 1 feedback.

To examine this issue, we employed cross-temporal decoding analysis^22,23^ (see “Methods”). We extracted cases in which a correct trial (T trial) in cycle 1 was immediately followed by the T trial of cycle 2, and concatenated these two trials. We labeled each case by a selected location in these two consecutive T trials. We then trained and tested the classifier using neuronal ensemble activity in all possible combinations of time windows ranging from the feedback onset in cycle 1 to the end of cycle 2 (1.3 s after feedback onset in cycle 2). The resultant classification accuracy indicates (1) strength of location coding in cases when the same time window was used for training and test (bins on main diagonal axis); and (2) similarity of population activity patterns between two different time points for off-diagonal bins.

In both PFC and inferior PPC, results in Fig. 4 showed that, during the Fb–Rw period in cycle 1, highly significant classification accuracy was observed along the main diagonal axis. Critically, however, there was no cross-generalization in population activity patterns between this task period and the beginning of the T trial in cycle 2, as indicated by prevalence of nonsignificant bins in plot areas at the intersection of these two task periods (arrows). This indicates that although the same target location was represented at the end of cycle 1 and the beginning of cycle 2, the underlying population activity patterns were remarkably dissimilar, and location preferences in cycle 1 were unrelated to preferences in a later cycle. In addition, even within cycle 2, population activity patterns were very different between the waiting period and the Fb–Rw period (see plot areas at the intersection of these two time periods).

The findings in Fig. 4 suggest distinctly different coding dynamics in PPC and PFC. In PFC, at the start of cycle 2, the period of peak location code in PEV indicated a transition from a first stable code, seen prior to and immediately following wait start, to a second stable code, arising later in the wait period and maintained up to the time of key release (Fig. 4a). In PPC, location coding was only strong at the time of the peak (Fig. 4b). The result relates to generally stronger location coding, before and after wait start, in PFC compared to PPC (Fig. 3a, b), suggesting somewhat different roles in target reactivation.

To complement this analysis, we repeated the same analysis over neural activities in PFC obtained in all periods of two consecutive T trials across cycles 1 and 2 (Supplementary Fig. S2a) and across cycles 2 and 3 (Supplementary Fig. S2b). The result showed two notable trends. First, not only between cycles 1 and 2 (Fig. 3a), but also between cycles 2 and 3 which both belonged to the memory-guided phase (Supplementary Fig. S2b), despite the fact that the same target information was represented across trials, there was no cross-generalization in location codes between the Fb–Rw period in a first trial and the beginning of a second trial. This result further reinforces the view that during an ITI between cycles, target information was not maintained in a stable, sustained code from one cycle to another. Second, near the wait-start time, there was a remarkable difference in the degree of cross-generalization of location codes between cycles (trials) (Supplementary Fig. S2c). That is, while between cycles 1 and 2, the location codes showed poor cross-generalization (see the diagonal axis between outlined characters A and B in the color map, Supplementary Fig. S2a), contrarily between cycles 2 and 3, the location codes showed a significantly greater degree of cross-generalization (see the diagonal axis between outlined characters A’ and B’ in Supplementary Fig. S2b). This result corroborates the results obtained in PEV (Fig. 3), suggesting different location codes in search (cycle 1) and memory-guided phases of the problem. Once the target memory was acquired in the Fb–Rw period in cycle 1, it underwent cycles of silencing and reactivation in the subsequent memory-guided phase, with comparable population codes arising afresh shortly after the onset of the wait start of a new trial and declining by around 500 ms.

These results provide an initial indication that memory for target location was not maintained in a stable population activity pattern, beginning when the target location was learned and continuing into subsequent trials. Instead, a novel target representation appeared at the beginning of a new trial, with a sudden increase in location coding, and a population activity pattern uncorrelated to the pattern seen during the processing of feedback.

### Similar wait-period coding in sequential memory and delayed-response tasks

Our analyses to date show that, in cycles 2–4 of the sequential memory task, a spatial code was implemented afresh at the start of each trial. We wondered how this retrieved code might relate to a spatial code in a conventional delayed-response task. At the same time we wished to replicate our results without confound between placeholder onset and the animal’s location choice on the first trial of cycle 1, allowing these trials to be retained in the analysis.

To address this, we trained a third, naïve monkey (monkey C) in a classic memory-guided saccade (MGS) task and an oculomotor version of the sequential memory task (one-target problems only) whose rules and displays were the same as in the original task, except for some minor modifications (see “Methods”). In this version, in particular, the placeholders were presented afresh at the beginning of every trial, which ruled out the possibility that any particular location(s) preferred by the monkey at the beginning of the search (cycle 1) was associated with visual response elicited by the placeholder presentation. The monkey’s performance in this task was near optimal. Mean numbers of trials to complete cycles 1, 2, 3, and 4 were 3.48 ± 0.32, 1.17 ± 0.16, 1.09 ± 0.05, and 1.08 ± 0.06, respectively (mean ± s.d.). We recorded 180 dorsal and ventral PFC cells in 12 sessions (recording locations, Supplementary Fig. S3).

Time series of population PEV showed that reemergence of target information in cycles 2–4 was replicated in the oculomotor sequential memory task (Fig. 5a). As in the original task, in cycle 1, location encoding rapidly waned before the end of trial (blue and red curves). Subsequently, in the wait period in cycles 2–4 (green curves), significant target information reemerged, exhibiting significantly greater PEV than in cycle 1 (black curve). In the MGS task (Fig. 5b), the same neural population showed a typical pattern of PEV time-course as reported in previous studies (e.g., ref. ^7^). Population PEV for selected location in correct trials showed an abrupt, phasic increase upon the presentation of the memory cue, remained significantly above zero during the delay, and increased again during the execution of saccades.

To complement the analysis in Fig. 4 and Supplementary Fig. S2, we performed the same cross-temporal decoding analysis over neural activities obtained in all periods of two consecutive T trials across cycles 1 and 2 (Supplementary Fig. S4a) and across cycles 2 and 3 (Supplementary Fig. S4b). The results observed in the original task were replicated in this oculomotor sequential memory task, except that near the wait-start time, the degree of cross-generalization of location codes between cycle 1 and cycle 2 was somewhat greater in the oculomotor task than in the original task (compare the diagonal axis between outlined characters A and B in the color maps in Supplementary Figs. S2a and S4a). This is likely because in the oculomotor task, both the motor preparation component common to cycles 1 and 2 and the target location memory specific to cycle 2 were coded in a retino-centered coordinate system, while in the original task, there were two reference frames, hand-centered and retino-centered coordinates, involved in motor preparation and location memory, respectively.

In addition, as shown in Supplementary Fig. S5, in the wait period of the oculomotor sequential memory task, the comparison of PEV among individual cycles gave a result comparable to that observed in the original task (Fig. 3c, d); cycle 2 showed the greatest increase in PEV as compared to cycle 1, and after cycle 2 there was a monotonic decrease in PEV toward cycle 4. Note that in the oculomotor task, there is a slight delay in peak time of the PEV time series as compared to the original task (Supplementary Fig. S5a), and the aforementioned result was best observed in a shifted analysis time window (300–800 ms from the wait-start time, Supplementary Fig. S5b). As in the original task, there is a clear and consistent decreasing trend in cycle-by-cycle PEV values across cycles 2–4.

To examine which aspect of the MGS activity related to the reemergence of target information in the sequential memory task, we again used a cross-temporal decoding approach. We examined whether a classifier that is trained to discriminate locations of the saccade target (memory cue) in MGS can do so when tested on the data recorded in cycles 2–4 of the sequential memory task. Here, we aimed to find out which MGS task period showed population activity patterns that were similar to the wait period in cycles 2–4 of the sequential memory task.

The result in Fig. 5c showed that in the wait period of the sequential memory task (gray shaded area), significant classification accuracy was observed when the classifier was trained in the cue and the subsequent delay periods in the MGS task. Confirming the absence of sustained location coding between trials of the sequential memory task, significant classification only arose around 250 ms prior to wait start, and was then maintained throughout the delay period. Thus, the population activity pattern in the wait period in cycles 2–4 had significantly above-chance similarity with the activity pattern elicited following the presentation of a memory cue in the MGS task. This suggests that there is a significant overlap between the neural processes underlying encoding of an exogenous cue which indicates a current goal and that underlying internal activation (reactivation) of goal information from memory.

Conversely, when we applied the classifier from MGS on the data recorded in cycle 1 of the sequential memory task (search phase), classification accuracy during the wait period was reduced (Fig. 5d). This result rules out the possibility that components unrelated to memory such as motor preparation are the sources of the observed significant representational similarity between the wait-period activity in cycles 2–4, and cue and delay activities in MGS (i.e., the vertical 0.5-s long strip in the color map following the wait start, which was between the two dotted vertical lines, Fig. 5c). The direct comparison of classification accuracies in these two sets of decoding analyses (Fig. 5e, f) revealed significantly greater similarity in location codes between the wait-period activity in cycles 2–4 and the cue and delay activities in MGS than between the wait-period activity in cycles 1 and the cue and delay activities in MGS (see the vertical 0.5-s long strip in the 2-D color map following the wait start in Fig. 5f). This result further reinforces our observation in Fig. 5c, d.

Overall, the results in one-target problems of the sequential memory task speak against a model of memory-guided target selection mediated by an unbroken chain of sustained activity from the feedback instruction from a preceding cycle. Instead, the representation of target location could be silenced after initial learning, but reactivated among frontal and parietal neural populations on later trials, implying a neural processes that self-generates an instruction signal based on memory. The present results also shed light on the relations between the sustained firing account and the activity-silent account of WM. The same PFC neuronal population exhibited both types of WM codes depending on the task setting, suggesting that memory reactivation, a key component of activity-silent WM, can be followed by sustained memory representation as usually observed in the standard MGS paradigm.

### Diminished signal of target reactivation in two-target problems

The analyses so far have focused on the one-target problems, in which the monkeys had to maintain only one target for the duration of each problem. In two-target problems, two targets were to be learned, maintained and selected on successive trials. We turn now to the neural consequences of this increase in task complexity and load.

Within each cycle, we term the first target selected T1 and the second T2. For our first analysis, as before, we calculated population-averaged PEVs with a factor of selected location. For cycle 1, we measured PEV separately for trials up to and including selection of T1 (termed “until T1” trials), and for trials after T1 up to and including selection of T2 (“until T2”). For cycles 2–4, only T1 and T2 trials (i.e., correct trials) were used. In broad outline, PEV plots resembled those of the one-target problems, with above-chance location coding in the wait period, especially in PFC, a rapid increase around key release, and rapid waning after reward (Fig. 6a, dorsal and ventral PFC cells; Fig. 6b, inferior PPC cells; Supplementary Fig. S6a, superior PPC cells). Again, location coding in the feedback period was stronger for cycle 1 than cycles 2–4. Conspicuously missing, however, was the signal of target reactivation at the start of the wait period in cycles 2–4. Instead, the strength of population coding for targets retrieved from memory remained close to the level seen in the search trials of cycle 1.

Although, in these two-target problems, the strength of location coding did not increase in retrieval (cycles 2–4) as compared to search trials (cycle 1), next we used decoding analysis to ask whether location representations themselves might change, as they did in one-target problems (Supplementary Figs. S2 and S4). We first focused on data from PFC, where location information was strongest. In a first analysis (the between-phase analysis), we examined how accurately a classifier trained using activity in cycle 1 (search phase) could decode activity in cycles 2–4 (memory-guided phase) and discriminate locations chosen on these trials. Results in Fig. 7a showed that, along the diagonal axis, there is a weak, gradually increasing trend in classification accuracy before the key-release time, which was followed by a phasic abrupt increase afterward. In a second analysis (the within-phase analysis), a classifier was trained using one-third of trials in cycles 2–4 and tested on the remaining trials in cycles 2–4. As compared to the between-phase analysis, the result showed higher classification accuracy before the key-release time, with similar, phasic peak afterward (Fig. 7b). Critically, comparison of the on-diagonal elements from the between-phase and the within-phase analyses indicated that, near the wait-start time, classification accuracy in the within-phase analysis gave significantly greater values than the between-phase analysis (Fig. 7c). This indicates that near the wait-start time, trials in cycles 2–4 (memory-guided phase) elicited some common population activity patterns that were not present in cycle 1 (search phase). This result was very weak in inferior PPC (Fig. 7d–f), and absent in superior PPC (Supplementary Fig. S7g–i).

There is a possibility that this difference in classification accuracies came from the difference in trial sampling methods; in the between-phase analysis, training and test trials came from different cycles while in the within-phase analysis, both training and test trials came from cycles 2–4. To exclude this possibility, we repeated the within-phase analysis with a modified trial sampling method. We first used cycle 2 to construct a classifier, and tested this classifier by using trials in cycles 3–4 to compute classification accuracy. We then repeated this procedure for the remaining two grouping patterns: cycle 3 vs. cycles 2 and 4; cycle 4 vs. cycles 2–3. The mean of the three classification results was regarded as the classification accuracy of the between-phase condition. With this procedure (Supplementary Fig. S7), we observed an almost identical result to the original version of this analysis, ruling out the possibility that the observed difference in classification accuracies between the two analyses in Fig. 7c can be attributed to the difference in trial sampling methods.

Compared to one-target problems, accordingly, two-target problems showed little evidence for a peak of location coding at the start of memory-guided trials. As they did in the one-target problems, however, location representations changed in form between search and memory-guided phases. Again the data suggest that, at the start of each memory-guided trial, there was reconstruction of a retrieved target code.

### Strength of location coding changes with target order

Next, for prefrontal cells, we compared PEV time series between T1 and T2 trials, separately for cycle 1 (Fig. 6c, left) and cycles 2–4 (Fig. 6c, right). The first notable result arose in cycles 2–4 (Fig. 6c, right). For these cycles with known targets, PEV for T2 trials (dark green curves) was almost always greater than that for T1 trials (light green curves), with several periods of significant difference. A similar, weaker trend was seen in inferior (Fig. 6d, right) but not superior (Supplementary Fig. S6b, right) PPC cells. In PFC and inferior PPC cells, location coding for T1 was also significantly weaker than coding in the one-target problem (T trials) in cycles 2–4 (Fig. 6c, d, right, solid vs dashed light green curves). Plausibly, in cycles 2–4 (memory-guided phase), location coding for T1 was diminished because a remaining, second target (T2) was still held in memory for selection on a subsequent trial in the same cycle.

### Frontal activity encodes both current and non-current targets

To develop this point, we examined whether, in two-target problems, neural activities reflected not only the target selected in the current trial (current target), but also a target that was not currently being selected (non-current target). For this analysis, we classified the four cycles in each problem in two groups: non-perfect and perfect cycles. Perfect cycles were those completed with just two trials, with the remainder called non-perfect. In perfect cycles, selection of current and non-current target occurred in immediately adjacent trials. All cycle 1 cases were classified as non-perfect cycles (see “Methods“). Percentage of non-perfect and perfect cycles in each cycle were as follows: cycle 1, 100% and 0%; cycle 2, 33% and 67%; cycle 3, 17% and 83%; cycle 4, 15% and 85%.

In perfect cycles, to examine time-course of strength of location encoding for current and non-current targets, we performed two-way ANOVAs with factors current target and non-current target on firing rates in each sliding window (width, 100 ms; slide, 50 ms) (Eq. (2), “Methods”). We conducted this analysis separately for T1 and T2 trials. For T1 trials, the non-current target was called future (coding of T2 location on T1 trial). For T2 trials, the non-current target was called past (coding of T1 location on T2 trial) (see “Methods”).

The results indicated two notable trends. First, in all of the three populations (PFC cells, Fig. 8a; inferior PPC cells, Fig. 9a; superior PPC cells, Supplementary Fig. S8a), regardless of the ordinal position of target touch (T1 or T2), the current target (green curves) was almost always more strongly represented than past (blue) and future (magenta) targets. Second, significant representation (PEV > noise) of non-current targets was most strongly observed in T1 trials, and only in PFC cells (future target, magenta curves, Fig. 8a, left); PEV for a future target (T2) ramped up gradually from the wait-start time in the T1 trial, and continued to give significant values into the T2 trial, when this representation then turned to indicate current target (green curves, Fig. 8a, right). In PFC cells, in addition, at the start of the T2 trial, there was a significant but short-lived PEV for past target, a target which was just visited in the preceding T1 trial (cyan, Fig. 8a, right). A similar result was seen in superior PPC cells (Supplementary Fig. S8a, right). This representation of a past target, persisting from a preceding T1 trial, disappeared around the wait-start time in T2 trials, though in PFC cells, it continued to give PEV values slightly above zero. Except for these cases, information regarding the non-current target was absent across all of the three neural populations. We repeated the same analysis over the non-perfect cycles. None of the three areas showed significant representation (PEV > noise) of non-current targets (Supplementary Fig. S9).

These results indicate that PFC cells not only encoded the location of a target selected in a current trial, but also maintained future and past targets in a sequence of memory-guided behavior. Importantly, there is a distinct temporal gradient in the strength of encoding, such that a future target was more strongly encoded than a past target. The present results also highlight a division of labor between PFC and PPC, such that target selection for a distant trial, the one after the immediate trial, is more likely to be represented by PFC. In PFC, accordingly, reduced location coding for T1 (see Fig. 6c, right) is accompanied by allocation of neural resources to prospective coding of T2.

To further characterize the prefrontal contribution to the processing of a future target, we examined whether or not representation of T2 location across T1 and T2 trials relied on a stable population code that persisted throughout the entire duration of the cycle. In this analysis, we first concatenated each cell’s activity in successive sequences of T1 and T2 trials within a perfect cycle and constructed a “supertrial”. We then assigned T2 location as a class label for that supertrial. We tested how the classification accuracy changed over time during the entire duration of the supertrial, assessing the similarity of population codes for T2 location across time points.

Note that in 37 PFC cells (7.4% of recorded PFC cells), 11 inferior PPC cells (4.3%), and 25 superior PPC cells (7.9%), which were recorded in four sessions (out of 84 total sessions), there were not sufficient repetitions (*k* = 4) in one of the five target locations to perform cross-validation. To include these cells in the analysis, we relaxed the conditions for a perfect cycle only for cycle 2 in these cells, by allowing one error trial before the two consecutive T1 and T2 touches. This measure has moved 28 supertrials (0.4% of total supertrials recorded in cycle 2 in 84 sessions) that had been classified as non-perfect cycle to the perfect cycle. Qualitatively identical results were obtained without including these cells.

As shown in Fig. 8b, at the beginning of the T1 trial (i.e., before reward (Rw) time), significant classification accuracy was observed along the diagonal time bins but not in off-diagonal bins. This indicates that these population codes are time-specific: the same T2 target was represented over time, but in rapidly changing population activity patterns, which is a hallmark of dynamic coding^6^. Most critically, there was no cross-generalization between equivalent periods of the T1 and T2 trials; though T2 location was significantly encoded in both trials, this encoding was implemented in independent patterns of population activity.

Once the T1 trial was completed (i.e., from Rw time in T1), significant classification accuracy appeared in both on- and off-diagonal bins and persisted until the key-release time in T2 trials. This indicates that the same T2 target was represented over time as in early T1 trials, but now in highly similar population patterns across different time points, a hallmark of sustained coding. Critically, these results suggest that the two seemingly opposing schemes for mnemonic processing, dynamic coding and sustained coding, can co-exist in the same prefrontal population, and the prefrontal cortex adaptively switches between the two, depending on a current stage in a multistep task.

To complement this analysis, we examined the changes in population codes for T1 location (i.e., using T1 instead of T2 location as a class label) across successive sequences of T1 and T2 trials within a perfect cycle (Fig. 8c). In PFC, again, T1 identity was encoded throughout both T1 and T2 trials, in largely dynamic format. Again, there was no cross-generalization between equivalent periods of the T1 and T2 trial, showing that T1 identity was encoded in independent activity patterns across the two trials. We repeated the same analysis over the inferior PPC (Fig. 9b, c) and superior PPC cells (Supplementary Fig. S8b, c). Results were very weak in both areas.

## Discussion

In this study, we examined activities of frontal and parietal neurons in a complex location selection task. In each problem of this task, the monkeys were first required to search through the stimulus display and discover currently rewarded location(s). Subsequently, they were required to continue to perform efficient location selection based on this learned information throughout the duration of the problem. The present paradigm can be regarded as a typical working memory paradigm, because this task, especially the two-target problem, taxed not only the maintenance of, but also the manipulation of the information held in memory, as the animal used task rules to alternately recall the two memorized target locations (i.e., they began each cycle with either target, but then avoided re-selection until next cycle).

There are four main findings of interest. (i) Over the course of problem-solving, WM for current targets showed substantial transitions in activity state. In both PFC and PPC, mnemonic representations for target locations were weak between trials, then reactivated at trial onset. The reactivated location code in cycle 2– 4 differed in form from the code for equivalent periods of a cycle 1 trial, when the location selection was not based on memory. (ii) In both regions, comparing one- and two-target tasks showed the effects of task complexity; in two-target problems, there was reduced reactivation of the target representation at the start of each trial. (iii) In simple problems, response properties were similar in PFC and inferior PPC, though in the wait period of each trial, codes were stronger and more sustained in the PFC. With increased problem complexity, only PFC carried a full task representation, encoding not only current but also past and future events. (iv) Accompanying silencing and reactivation, PFC codes for the same target information also transformed across trials. Past, current and future events within a sequence of behavior were encoded in uncorrelated, largely dynamic activity patterns, with limited capacity to encode a current event when a second, future event was also to be maintained. These topics are discussed in turn below.

In this study, we did not observe sustained representations for target location that persisted throughout the duration of a problem, nor even between cycles, as a source of information to guide location selection in each trial. Instead, in both PFC and inferior PPC, target memory rapidly reduced at the end of each trial and was reactivated afresh at the start of a new trial, even without any external instruction cues. Evidence for this came from a number of converging sources. In one-target problems, location information peaked shortly after trial onset (Figs. 3 and 5), corresponding to a switch from dynamic to stable location coding (Supplementary Figs. S2 and S4). In two-target problems, while the location of a current target was strongly encoded (Fig. 6), neural activity contained much less (PFC) or no (PPC) information concerning a second target, selected on a preceding or following trial (Figs. 8 and 9).

In conventional delayed-response paradigms, sustained, stable location coding has often been found to extend from initial target presentation to the time of response (for discussion, see refs. ^4^,^9^). In more complex tasks, calling for locations to be stored across successive trials, a few prior studies of prefrontal cortex have reported observations similar to ours. For example, in a dual-task comprised of two spatial delay tasks, when the interleaved task is terminated, the main-task codes that had been outside the focus of attention are reactivated prior to the behavioral response for the main task^7,24^. Similarly, Barbosa et al. showed that in the standard MGS task, although information about the target location in a trial disappeared soon after the completion of that trial, it was silently maintained across ITI by spike synchrony selective to the previous stimulus^25^. This information was reactivated just before the start of the next trial, and enhanced serial biases in location memory, small systematic shifts of memory-guided saccades toward a location memorized in the previous trial. Similar results were obtained in human neuroimaging studies which reported temporary loss and later reestablishment of significant classification accuracy for memorized objects in multivariate pattern analysis^5,26,27^.

Our results corroborate these studies and extend them in two important points. In our study, memory reactivation exhibited different forms, depending on task setting. Reactivation was most prominently observed in the one-target task (Figs. 3 and 5). In the two-target task, the trend became less conspicuous in PEV time series (Fig. 6), though decoding analysis confirmed that, after initial learning, the newly formed memories for target locations were reactivated at the start of new trials (Fig. 7c and Supplementary Fig. S7c). In PFC only, at the start of a perfect cycle, not only a memory of the current target, but also a memory of the future target were reactivated (Fig. 8a). We suggest that in many situations, WM processing in the frontoparietal network may not depend on sustained codes. Coding of task contents by silencing and reactivation of related neural activity appears to be a widespread phenomenon of WM processing.

Second, we showed how reactivation is related to neural activities in a classical delayed-response paradigm (Fig. 5)^1,2,28^. In PFC, classifiers trained on the cue and delay activity in the MGS task could successfully decode the reactivation-related activity near the wait-start time in the sequential memory task. Such cross-generalization from the MGS task arose shortly before trial start, adding further evidence for memory reactivation (Fig. 5c). This could only be possible if external shift of attention to a spatial cue in MGS involved similar neural processes as an internal shift of attention to a location currently being held in memory. This suggests that, even without external instructions, prefrontal cells can self-generate the necessary information from recent memory and make it an instruction signal to guide forthcoming behavior. It is also worth noting that, while the reactivation phenomenon observed in Barbosa et al. corresponded to the storage of passive short-term memory traces that were not actively used in a subsequent trial^25^, in our study the reactivated information clearly contributed to goal selection in the ensuing trial (Fig. 5c). Of course, the present results do not rule out the possibility that there are sustained codes in other areas that send information to the reactivation cells we recorded in PFC and PPC^29^. For example, there is a possibility that the medial temporal lobe memory system supported retention of target information^30,31^.

Previously, in a pioneering study of prefrontal mechanisms underlying self-organized behavior, Procyk and Goldman-Rakic used a spatial problem-solving task similar to our one-target task^21^. They compared prefrontal neuronal activity between this task and a standard delayed-response task, and reported that prefrontal neurons which showed location-selective delay activity in the delayed-response task (i.e., delay neurons) also encoded a known target location prior to behavioral response in the problem-solving task, with similar location preference between the two tasks. They also reported that in the problem-solving task, the representation for a known target temporally disappeared between different trials (i.e., cycles in our task). The authors, however, had suggested that the observed successful information maintenance without sustained code was due to difference in task demands: because working memory was less heavily engaged in the memory-guided phase of the problem-solving task, compared to the delayed-response task, sustained activation which carried target information was strongly attenuated in the problem-solving task. In the present study, while we replicated their key observations in the one-target task, as we have discussed, several lines of evidence, including direct demonstration of relations between neural activity in the MGS task and the sequential problem-solving task (Fig. 5), indicate that the reappearance of a known target code at the beginning of new trials corresponds to reactivated target signal from a latent storage state.

While our data show strong transitions in the two-target task, with selective coding of the target location selected in the current trial, this transition was complete only in the PPC. In PFC, several features of the results indicate a more complete representation of events across a whole two-target cycle. Most strongly, there was future coding, with a population representation of T2 location extending through the T1 trial. A similar result was reported in a prior study which used a serial self-ordered search task, in which within an array of six spatial targets, monkeys were allowed to select the targets in any order^32^. The authors showed that PFC cells not only encoded the location of the current target but also that up to several steps away. The present study gives several novel insights. First, future codes, while not within the focus of attention (i.e., during T1 trials), gradually ramped up behind the current target code which was attentionally prioritized. Upon the completion of target selection in T1 trials (i.e., after reward time), future codes then took over the current code and became a dominant representation (Fig. 8a). In PFC there was also a weaker retrospective code, continuing to signal the T1 location during the T2 trial. Thus, increases and decreases in strength of encoding for each of the two targets were determined by attentional states. These results provide direct neurophysiological support for the model which considers working memory as attentional selection (internal activation) from a subset of representations already being held in memory^33–35^.

Second, PFC maintained past, current and future targets in independent activity patterns. During T1 trials of a perfect cycle, in addition to the strong representation of T1 location, there was dynamic representation of the planned T2 location. This representation, however, showed no cross-generalization to the T2 location signal in T2 trial itself, when the status of this target changed from future to current (Fig. 8b). A similar, weaker result held in reverse; during the T2 trial, accompanying the strong T2 representation, there was a retained code of T1 location (Fig. 8a, right), again dynamically varying over the T2 trial, and not cross-generalizing to the code of this same T1 location during the T1 trial itself (Fig. 8c). The results are reminiscent of changes in premotor information coding as the animal shifts from preparation to execution of a movement^36^. It has been argued that, during movement preparation, information is coded in an “output-null” space, ineffective in driving motor output. At the time the movement is made, the representation shifts into an “output-potent” space, resembling the shift in our task between coding for currently unselected and currently selected locations.

Despite independence of past, current and future target codes, there was evidence for interference between them. Prospective coding for T2 location in the T1 trial was stronger than retrospective coding of T1 location during the T2 trial (Fig. 8a, magenta vs light blue curves), and perhaps reflecting this asymmetry, the current target code was weaker in the T1 trial than in the T2 trial (Fig. 6c, right). Along similar lines, in both PFC and PPC, the sudden increase in location coding seen at the start of a one-target trial was reduced or absent with two targets in WM.

In monkeys, similar neuronal responses have often been observed for cells in the PFC and inferior PPC^11,37,38^. In the present study, while cells in PFC and inferior PPC showed largely similar response patterns, there were also several differences. In one-target problems, PFC more strongly represented target location before wait start, with transition to a new form when the wait period began and location coding showed a sharp increase (Figs. 3 and 4). Over successive cycles, location coding in the wait start period declined in PFC, but remained approximately stable in PPC (Fig. 3d). Differences between PFC and PPC were most marked, however, in two-target problems. While PPC coding focused only on events of the current trial, PFC coding was more comprehensive, encompassing past, current and future events within a larger behavioral program. While most of the reported differences in neuronal activity between PFC and PPC are quantitative (but see ref. ^39^), the present results suggest a qualitative difference. Taken together, these results suggest that the PPC code is more strongly driven by immediate events, while the PFC code reflects both the state of learning and the wider context of an entire behavioral program.

Overall, our data suggest that the timely silencing and reactivation of component action plans across frontoparietal cortices underlies adaptive structuring of self-organized behavior in complex problem-solving. Within the frontoparietal region, there is a division of labor. Both dorsal and ventral PFC carried a full task representation, encoding not only current but also past and future events, and adaptively engaged in silencing and reactivation of goal representations, even when the task involved multiple steps. Inferior PPC, on the other hand, was primarily concerned with reactivation of the immediate goal representation, irrespective of task complexity. Superior PPC was only involved in the sensorimotor aspect of the task. The present results provide compelling neuronal evidence for transitions in activity state within WM. They suggest how PFC and PPC collaborate to create successive cycles of attentional foregrounding and backgrounding in complex behavior.

## Methods

### Experimental design

#### Experimental model and subject details

In the main experiment, we used two male rhesus monkeys (*Macaca mulatta*, monkeys A and B), each weighing 13 kg. The experiments were conducted in accordance with the Animals (Scientific Procedures) Act 1986 of the UK; all procedures were licensed by a Home Office Project License obtained after review by Oxford University’s Animal Care and Ethical Review committee. In the additional experiment (Fig. 5), we used one female Japanese monkey (*Macaca fuscata*, monkey C), weighing 8.0 kg. The experimental procedures were approved by the Animal Research Committee at the Graduate School of Frontier Biosciences, Osaka University and were in full compliance with the guidelines of the National BioResource Project “Japanese Macaques”.

### Behavioral task

In the main experiment, events in the sequential memory task were controlled by a REX system^40^, with displays presented on a 17.5 inch LED touchscreen placed in front of the monkey’s chair. A custom-made start key was attached to the front of the chair at monkey’s chest height.

Details of events on each trial are shown in Fig. 1a (bottom, “Example trial sequence”). Before the trial began, the screen showed an intertrial display (see below). To start a new trial, the monkey was required to hold down the start key, and to acquire and hold central fixation (a square window 7.6 × 7.6 ° visual angle). At this point, the FP turned red to indicate the start of a wait period. On trial immediately after the transition between cycles and problems (i.e., a first trial of a new cycle), an array of small square or circle placeholders (each 5.7 × 5.7 °, centered 11.4 ° from FP) reappeared at predetermined locations (18°, 90°, 162°, 234°, and 306° locations). On other trials (i.e., from second trial onward in each cycle), to indicate that the current cycle had not been completed, the placeholders were continuously presented on-screen as an intertrial display (i.e., the placeholders stayed on-screen throughout a cycle). The placeholders were removed from screen only between cycles and problems. To reinforce the monkey’s knowledge about when each cycle ended, the shape of placeholders was alternated between square and circle between successive problems.

The wait period continued for 1.2–2.0 s, termination of which led to change in the color of FP from red to cyan (Go). Fixation to FP was required throughout the wait period. The go signal indicated that a response could be made. To indicate his choice, the monkey released the home key (key release) and touched one of the placeholder locations. Touch was required within 1.8 s of GO. After the touch had been held for 0.35–0.45 s, the selected placeholder was replaced either by a green (target) or red (nontarget) square (feedback period, Fb), which remained for 0.3–0.4 s followed by an intertrial display. If the touched location was a target, a drop of soft food (reward, Rw) was delivered 0.05–0.15 s after Fb offset. If the selected location was a nontarget, there was a pause of the same duration as in reward delivery. Premature key release or fixation break prior to GO, or failure to hold the touch on selected location until the Fb signal, led to immediate termination of the trial without Rw.

Different intertrial displays indicated transitions within a cycle, between cycles, and between problems. For the transition of trials within a cycle, the intertrial display was the white FP and the placeholder array, with a minimum wait interval of 1.3–1.5 s required before the next trial would begin. For trials between cycles, the intertrial display was the white FP only, and lasted 3.3–3.6 s. To indicate the end of a problem, the screen went blank for 3.3–3.6 s, followed by a reappearance of the white FP, which prompted the monkey to initiate a first trial of a new problem by key press and central fixation.

In an additional experiment (Fig. 5), we compared activity of prefrontal cells between an oculomotor version of the sequential memory task and a standard memory-guided saccade (MGS) task. Task events were controlled by TEMPO experiment control system (Reflective Computing, WA), with displays presented on a 17 inch TFT monitor placed 50 cm from the monkey’s eyes.

In the oculomotor sequential memory task, rules, displays and order of events were the same as in the original task except that, (i) only one-target problems were presented, (ii) the monkey used saccadic eye movement to indicate choice, and (iii) the placeholders were removed from screen after completion of every trial and put back on at the beginning of a next trial. Before the trial began, a blank intertrial screen was presented, which lasted 1.0–1.2 s for transition of trials within a cycle, 2.4–2.8 s for between cycles, and 8.0–9.0 s for between problems. Subsequently, a white central FP appeared, and the monkey initiated a new trial by fixating the FP. After a brief fixation interval (0.15–0.25 s), as in the original task, an array of five small square or circle placeholders (each 3.5 × 3.5 deg, centered 12 deg from FP) were presented at predetermined locations, which marked the start of a wait period. The wait period continued for 1.2–2.0 s, termination of which led to the disappearance of FP (GO signal). To indicate her choice, the monkey was required to make a saccade to one of the placeholders within 0.55 s and hold fixation for 0.35–0.45 s. The selected placeholder was then replaced either by a green (target) or red (nontarget) patch (Fb), which remained on-screen for 0.35–0.45 s. Subsequently, if the selected location was a target, a drop of juice (Rw) was delivered for 0.15 s. If the selected location was a nontarget, there was pause of 0.15 s. The screen went blank after Rw. Fixation break prior to GO, or failure to hold the selected placeholder led to immediate termination of the trial without Rw.

In the MGS task, after a fixation interval (1.0–2.5 s), a small square was briefly presented (0.25 s) at one of the same five peripheral locations used in the sequential memory task. After a variable delay (1.5–4.0 s), the FP was replaced by small placeholders (square, 0.5 × 0.5 °) presented at all five possible cue locations. The monkey was required to make a saccadic eye movement within 0.55 s to the location where the visual cue had been presented and hold fixation for 0.35–0.45 s, after which the screen went blank and reward was delivered without any visual feedback. The sequential memory task and the MGS task were performed in separate alternating blocks with each block lasted for 80–140 and 40–60 correct trials, respectively.

We collected and analyzed behavioral and neural data in a total of 96 daily sessions (44, 40, and 12 sessions in monkeys A, B, and C, respectively).

### Neural recording

For monkeys A and B, under general anesthesia, we sterotaxically implanted a titanium head holder and recording chambers (Gray Matter Research) on the skull. Frontal chambers were placed over the lateral prefrontal cortex of the right hemisphere in both monkey A (AP = 39.9, ML = 20.3; AP, anterior-posterior; ML, medial-lateral) and monkey B (AP = 36.2, ML = 58.1). Posterior chambers were placed over the right parietal cortex in both monkey A (AP = −4.6, ML = 50.6) and monkey B (AP = −3.2, ML = 47.4). A 32-channel semichronic Microdrive was mounted inside each chamber. Recording locations in each monkey are shown in Fig. 1c.

We recorded neural data using 32-channel semichronic microdrive systems (SC-32, Gray Matter Research) mounted inside each chamber. The microdrive housed 32 individually movable single-contact tungsten electrodes with interelectrode spacing of 1.5 mm, and was interfaced to a multichannel data acquisition system (Cerebus System, Blackrock Microsystems). Raw extracellular signals were amplified, filtered (300 Hz to 10 kHz), and recorded in reference to the titanium head holder for offline sorting (Offline Sorter, Plexon). Between sessions, to ensure recording of new cells, electrodes were advanced by a minimum of 62.5 μm. Eye movement data were sampled at 120 Hz using an infrared eye-tracking system (Applied Science Laboratories) and stored for offline analysis. We did not preselect neurons for task-related activities; instead, we advanced microelectrodes until we could isolate neuronal activity before starting the task.

For monkey C, during both training and neural recording sessions, we used a non-invasive head-restraint method, using a thermoplastic head cap^41^. This head cap is made of a standard thermoplastic sprint material (MTAPU, 3.2 mm thick, CIVCO Radiotherapy, IA), and was molded out so that it conformed to the contours of the animal’ scalp (skull), cheek bone and occipital ridge. We stereotypically implanted a plastic cuboid recording chamber (width 12 mm, depth 16 mm, height, 15 mm, S-company ltd., Tokyo, Japan) on the left lateral surface of the prefrontal cortex (Supplementary Fig. S3). We recorded neural data using 32-ch linear microelectrode arrays (Plexon U-Probe, Plexon, TX) with an interelectrode spacing of 150 µm. We positioned the U-Probe by using a custom-made grid (width 12 mm, depth 16 mm, height, 10 mm) which had a total of 165 holes with 1 mm spacing. We advanced the U-Probe by a custom-made hydraulic microdrive (S-company ltd.). Raw extracellular neural signals were amplified, filtered (300 Hz to 6 kHz) and recorded in reference to the shaft of the linear array (monkey C) for offline sorting (Offline Sorter, Plexon) using a neural signal amplifier RZ2Bioamp Processor (Tucker-Davis Technologies, Fl). Eye movement data were sampled and stored at 120 Hz using an infrared eye-tracking system (ISCAN, MA).

At the end of the experiments, monkeys A and B were deeply anesthetized with barbiturate and then perfused through the heart with heparinized saline followed by 10% formaldehyde in saline. The brains were removed for histology and recording locations were confirmed.

### Statistical analysis

All statistical analyses were assessed by two-tailed tests, using MATLAB (MathWorks). To avoid multiplicity effect (an inflated Type I error in a family of hypothesis tests), we used Benjamini–Hochberg (BH) procedure to control the false discovery rate (FDR)^42^. We report FDR-controlled *P* values unless otherwise noted. All statistical analyses were assessed by two-tailed tests.

### Analysis of location selectivity in single-neuron activity

In this study, our main focus was to clarify at which stage of the sequential spatial memory task, the representation of target location(s) emerged among prefrontal and parietal populations, and how this representation was conveyed and evolved across successive task episodes to guide monkeys’ behavior.

To investigate neural coding of target representation, we calculated time series of each neuron’s proportion of explained variance (PEV) for all recorded neurons. For our main analyses (Figs. 3, 5, and 6), PEV was measured by the omega-squared (*ω*^2^) index of effect size in one-way ANOVA with a factor of selected location in the current trial. For cycles 2–4, only correct trials (i.e., T trials) were used. For cycle 1, both incorrect (NT) and correct (T) trials were used. Thus, except for NT trials in cycle 1, selected location corresponded to the target location defined in that problem. The *ω*^2^ PEV was calculated by the formula

1 $${\omega }^{2}=\frac{{{SS}}_{{{{{{\rm{effect}}}}}}}-\,{{df}}_{{{{{{\rm{effect}}}}}}}\times {MSE}}{{{SS}}_{{{{{{\rm{total}}}}}}}+{MSE}}$$

where *SS*_effect_ is the sum of squares between groups (locations), *df*_effect_ is the degree of freedom for the factor, location, *SS*_total_ is the total sum of squares for the entire data set, and *MSE* is the mean squared error within groups.

To match trial numbers when comparing PEV values between cycle 1 and cycles 2–4, we adopted the trial sub-sampling method reported in ref. ^39^ (Figs. 3, 5, and 6 and Supplementary Figs. S1 and S6). Specifically, for each neuron, we drew random subset of trials (equal to the number of trials in the smaller of the two groups, cycle 1 and cycles 2–4) from trials in the larger of the two groups, and calculated the *ω*^2^ statistic using these subsets of trials. For each neuron we repeated this process 25 times and used the mean of these values as the PEV estimate.

For time-series analysis, PEV was calculated in 100-ms time windows slid in increments of 50 ms. Within each time window, the observed PEV values were compared with noise level (PEV = 0), or PEV values in other conditions by permutation test. The resultant permutation p values were corrected for multiple comparisons (time windows) by controlling FDR under BH procedure. The significance level was set at *P* < 0.05.

### Cross-temporal decoding analysis

To evaluate the stability of location representations, we employed a cross-temporal decoding method as implemented in the neural decoding toolbox^43^. For each recording region, we pooled activities of all recorded neurons to create a pseudo-population. The firing rate of each neuron (calculated in 100-ms window slid in 50 ms) was z-score normalized in each time window.

We used a maximum correlation coefficient classifier (MCC) in *k*-fold leave-one-out cross-validation (*k* = 5, 10, 4, 4, and 4 trials for the results presented in Figs. 4, 5, 7, 8, and 9, respectively). Specifically, for each neuron and each time window, we first randomly sampled *k* trials, and concatenating across neurons, created *k* pseudo-population vectors for each class (location). We used *k* − 1 of these as training set. That is, for each class, we took *k* − 1 of the pseudo-population vectors and average them to create one prototype vector. We created a total of five prototype vectors corresponding to five selected locations. Next, we used the remaining “left-out” pseudo-population vectors (i.e., test trial; one vector per class), and calculated Pearson’s correlation coefficient with each of the five prototype vectors obtained from the training set. A class with the highest correlation coefficient was returned as the predicted label for that test trial. We divided the number of correct predictions by the number of classes (i.e., five) to compute classification accuracy.

We repeated this process *k* times, leaving out a different test pseudo-population vector each time and training the other *k* − 1 vectors to create the prototypes. We averaged the resultant *k* classification accuracy values to obtain an estimate of the classification accuracy for that time window for this iteration. The whole process was reiterated for 80 times (resampling runs) and the results averaged to give a final classification accuracy result.

Each prototype vector created in training trials was tested at both the same and different time windows. The result of the cross-temporal decoding analysis was presented as a color heat map of average classification accuracy at every combination of training and testing time windows.

### Analysis on representation of past, current, and future targets

In multiple target problems, there are three kinds of location information that need to be processed: information for a target being selected in the current trial (current target), that for a target that had been selected before the current trial (past target), and that for a target the monkey planned to select after the current trial (future target). To examine their neural representations (Figs. 8 and 9), we performed two-way ANOVAs with factors current target and non-current target on firing rates in each sliding window (width, 100 ms; slide, 50 ms). As the two location factors were not completely crossed, these ANOVAs only gave only main effects but not interactions. We conducted this analysis separately for two kinds of trials in which current target corresponded to a first or a second target touch in that cycle (T1 and T2 trials, respectively). In T1 trials, the non-current target corresponded to a target that would be visited in T2 trials (future target). In T2 trials, the non-current target corresponded to a target that had already been visited in T1 trials (past target). We used a partial *ω*^2^ PEV to measure both the strength of representation for current target and that for the non-current target. The partial *ω*^2^ PEV was calculated by the formula

2 $${\omega }_{p}^{2}=\frac{{{df}}_{{{{{{\rm{effect}}}}}}}({F}_{{{{{{\rm{effect}}}}}}}-1)}{{{df}}_{{{{{{\rm{effect}}}}}}}\left({F}_{{{{{{\rm{effect}}}}}}}-1\right)\times N}$$

where *df*_effect_ is the degree of freedom, *F*_effect_ is the *F* value of the factor under consideration, and *N* is the total number of trials. The partial *ω*^2^ is independent of other factors in the design^44^.

We repeated this entire process separately for trials in “non-perfect cycles” and those in “perfect cycles”. We classified the four cycles in each problem in two groups: non-perfect and perfect cycles. We classified a cycle as non-perfect cycle which was not completed with two trials, and that as perfect cycle which was completed with two trials (i.e., by two successive target touches). All cycle 1 cases were classified as non-perfect cycle, because by the task design, completion of cycle 1 in two trials can only happen by coincidence.

### Reporting summary

Further information on research design is available in the Nature Portfolio Reporting Summary linked to this article.

### Supplementary information

Supplementary Information

Reporting Summary

### Source data

Source Data

## Data Availability

The raw data that support the findings of this study are openly available in the G-node GIN repository at https://gin.g-node.org/KeiWatanabe/Watanabe_Kadohisa_Kusunoki_Buckley_Duncan.git. Source data are provided with this paper.

## Code availability

The codes to generate the main figures and results are openly available at the same URL as indicated in “Data availability”. The lead contact of this study is Kei Watanabe. Further information and requests for other resources related to this study should be directed to and will be fulfilled by the lead contact (kei_watanabe@fbs.osaka-u.ac.jp).
