# Supplementary material for: Cycles of goal silencing and reactivation underlie complex problem-solving in primate frontal and parietal cortex
Source: Nat Commun. 2023 Aug 19;14:5054. doi: 10.1038/s41467-023-40676-1 (PMC10439911; doi:10.1038/s41467-023-40676-1)
Supplement: Supplementary file 1 — Supplementary Information [file 41467_2023_40676_MOESM1_ESM.pdf]

Supplementary Materials for

Cycles of goal silencing and reactivation underlie complex problem-solving in primate frontal and parietal cortex

Kei Watanabe, Mikiko Kadohisa, Makoto Kusunoki, Mark J. Buckley, John Duncan

* Correspondence to: kei_watanabe@fbs.osaka-u.ac.jp

This PDF file includes:

Supplementary Figures S1 to S9

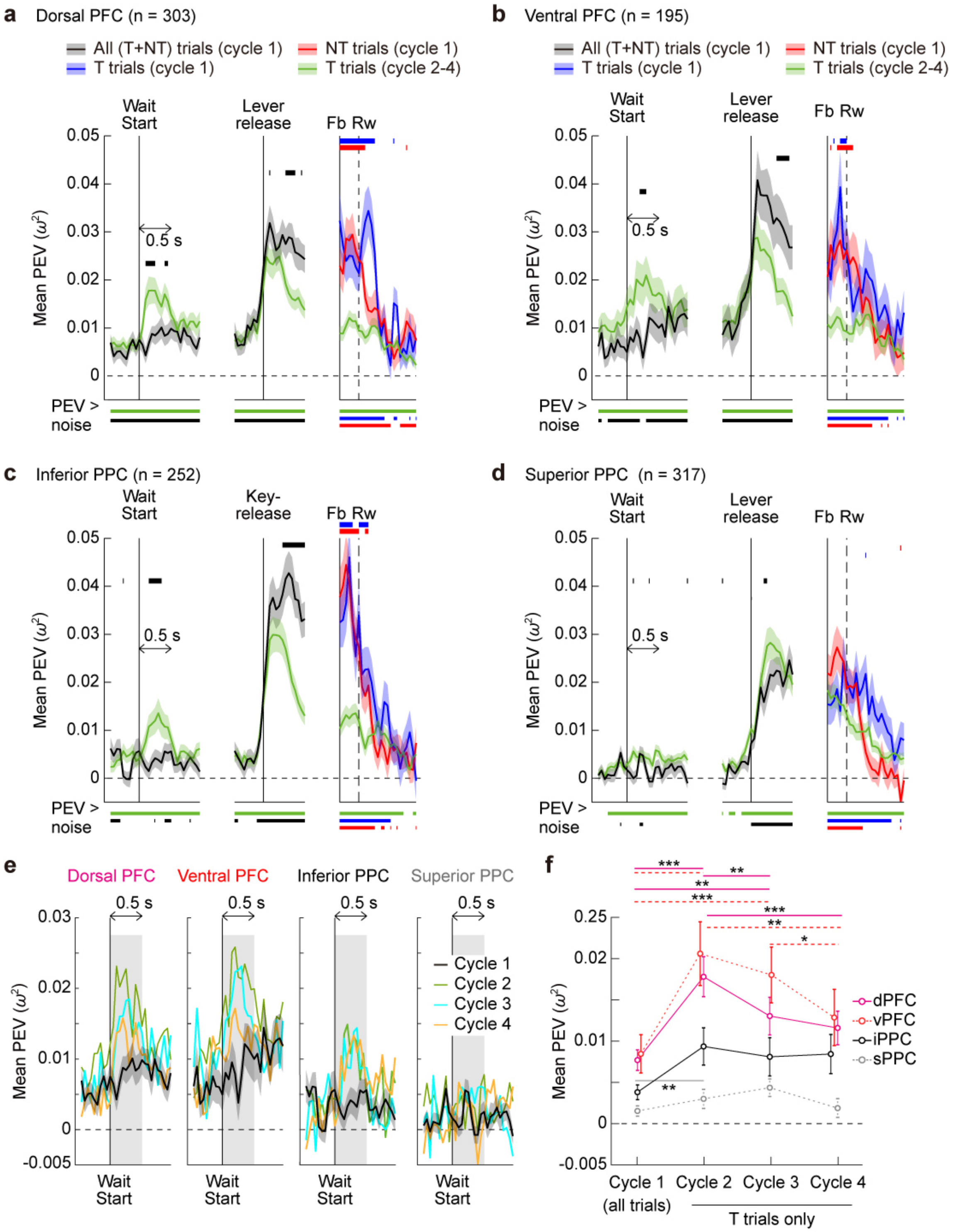

Figure S1. Location selectivity in the one-target task in each recording area. (**a-d**) Time-series of mean PEV (ω^2) for location over all recorded cells in the dorsal PFC (n = 303) (**a**), ventral PFC (n = 195) (**b**), inferior PPC (n = 252) (**c**), and superior PPC (n = 317) (**d**). Shaded areas indicate SEM. (**e**) Time-series of PEV before and after the wait-start time for each individual cycle in the four brain regions. (**f**) Comparison of mean PEV in the wait period (shaded area in **e**) across cycles in the four brain regions.

Error bars indicate SEM. A 4-by-4 two-way mixed-design ANOVA with factors area and cycle showed PEV in the wait period differed significantly depending on area and cycle (main effect of area, $F_{3, 1063}$ = 10.7, $P < 10^{-4}$; cycle, $F_{3, 3189}$ = 19.5, $P < 10^{-4}$; interaction, $F_{9, 3189}$ = 2.4, P = 0.01). Post-hoc simple effect analyses confirmed that dorsal and ventral prefrontal cells showed highly significant effects of cycle ($F_{3, 3189}$ > 8.7, $P < 10^{-4}$), with almost identical patterns across cycles. Inferior parietal cells showed a more moderate trend ($F_{3, 3189}$ = 3.1, P = 0.03). In superior parietal cells, the effect of cycle did not reach significance ($F_{3, 3189}$ = 0.8, P = 0.5). Asterisks indicate significantly different comparisons in the *post-hoc* simple effect ANOVA (* $P < 0.05$, ** $P < 0.01$, *** $P < 0.001$). dPFC: dorsal PFC; vPFC: ventral PFC; iPPC: inferior PPC; sPPC: superior PPC. Other conventions as **Figure 3**. Source data are provided as a Source Data file.

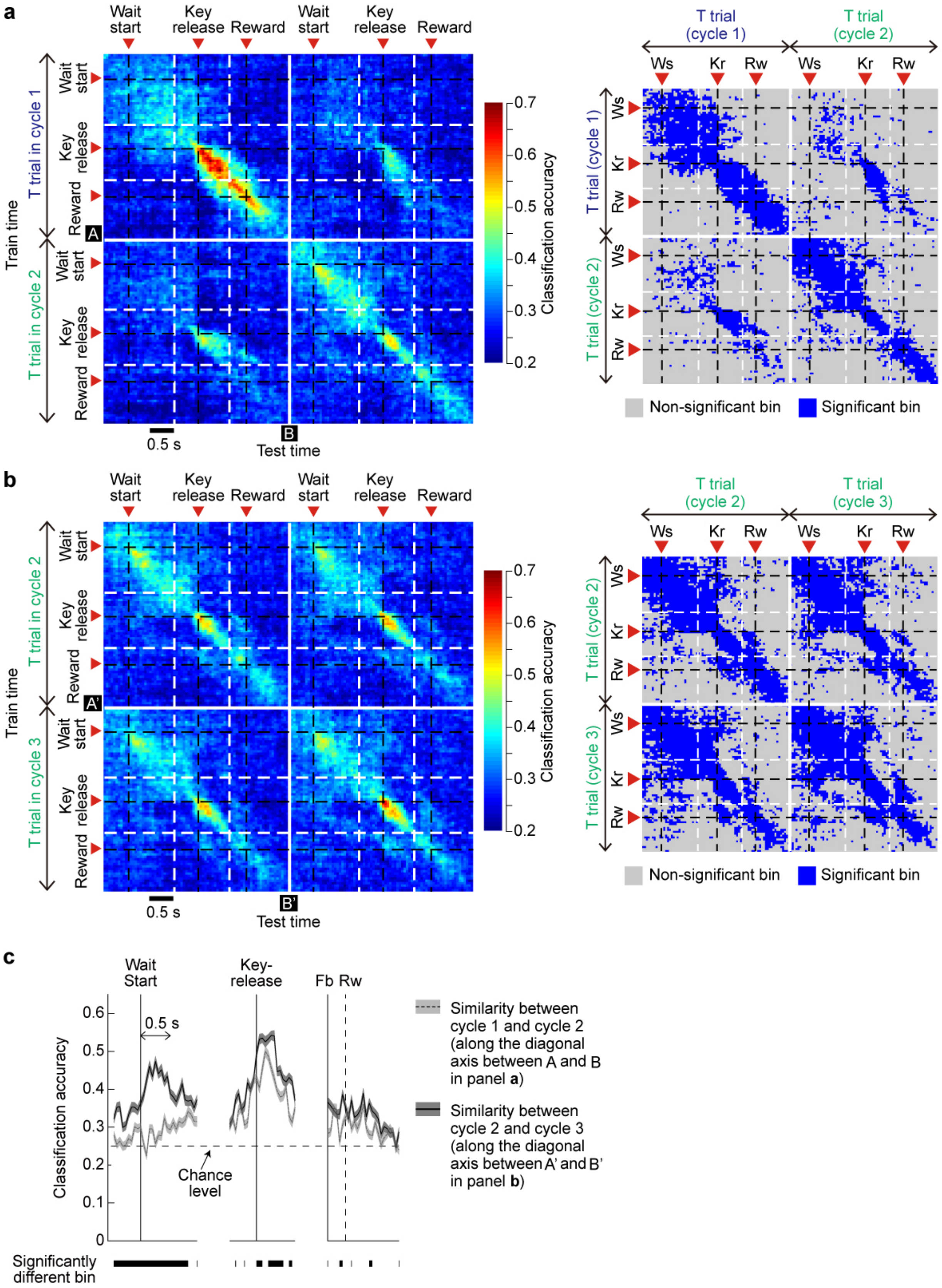
a
Wait start
Key release
Reward
Wait start
Key release
Reward
T trial in cycle 1
T trial in cycle 2
Train time
A
B
0.5 s
Test time
Classification accuracy
0.7
0.6
0.5
0.4
0.3
0.2
T trial (cycle 1)
T trial (cycle 2)
Ws
Kr
Rw
Non-significant bin
Significant bin
b
A'
B'
T trial in cycle 3
T trial (cycle 3)
c
Wait Start
Key-release
Fb Rw
0.5 s
Chance level
Significantly different bin
Similarity between cycle 1 and cycle 2 (along the diagonal axis between A and B in panel a)
Similarity between cycle 2 and cycle 3 (along the diagonal axis between A' and B' in panel b)

Figure S2. Dorsal and ventral PFC: cross-temporal decoding analysis in all periods of T trials across two consecutive cycles. (**a**) Results of cross-temporal decoding analysis across cycles 1 and 2. Conventions as in **Figure 4**. (**b**) Same as in **a**, but across cycles 2 and 3. (**c**) Difference in the degree of cross-generalization of location codes across cycles between panels **a** and **b**. Black curves indicate classification accuracies along the axis between outlined characters A and B in the color map in **a**. Dashed grey curves indicate classification accuracies along the axis between outlined characters A' and B' in the color map in **b**. Shaded areas indicate SEM. Lower horizontal bars indicate time bins with significant difference between the two curves (FDR-controlled permutation test, $p < 0.05$). Note that for the analysis in this figure only, after the removal of first trials of cycle 1 which was done to avoid the confound of visual response to placeholder (see Results), we have additionally removed the data for the 234° location (i.e., the analysis was performed using the remaining four locations). This is because after the removal of first trials of cycle 1, there were not enough trials in the 234° location in some neurons to perform a decoding analysis with the same *k*-fold cross validation as in the original analysis in **Figure 4**. No similar issue applied to the same analysis conducted on the oculomotor version of the task (see **Fig. S4**).

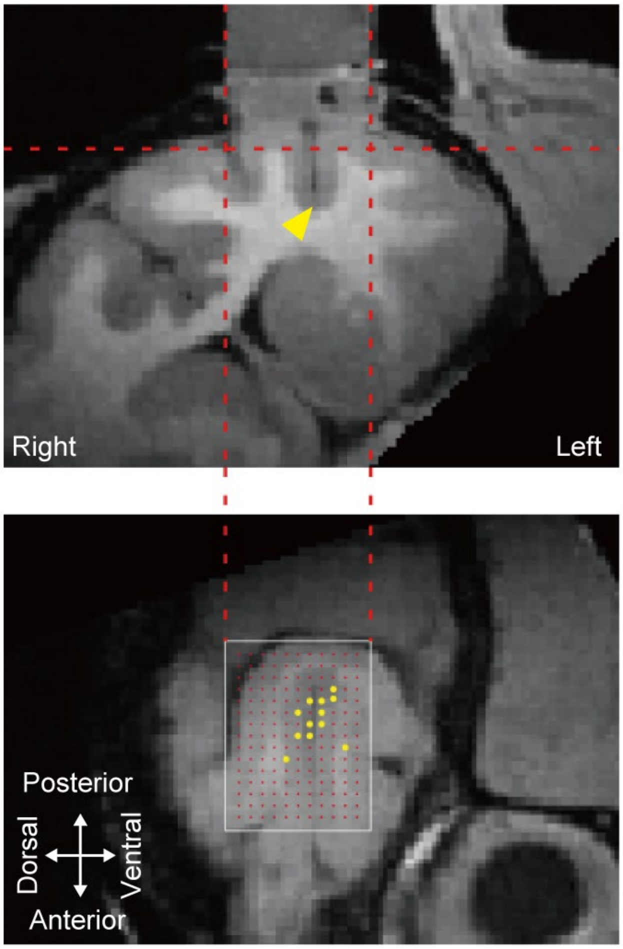

Figure S3. Recording locations for monkey C. In order to image the interior of the chamber, we filled the chamber with iodine solution. In the upper picture, the yellow arrow indicates the principal sulcus. In the bottom picture, locations of the 11-by-15 grid holes (red dots) were superimposed over the MR picture. Yellow dots indicate grid holes where we performed neural recording.

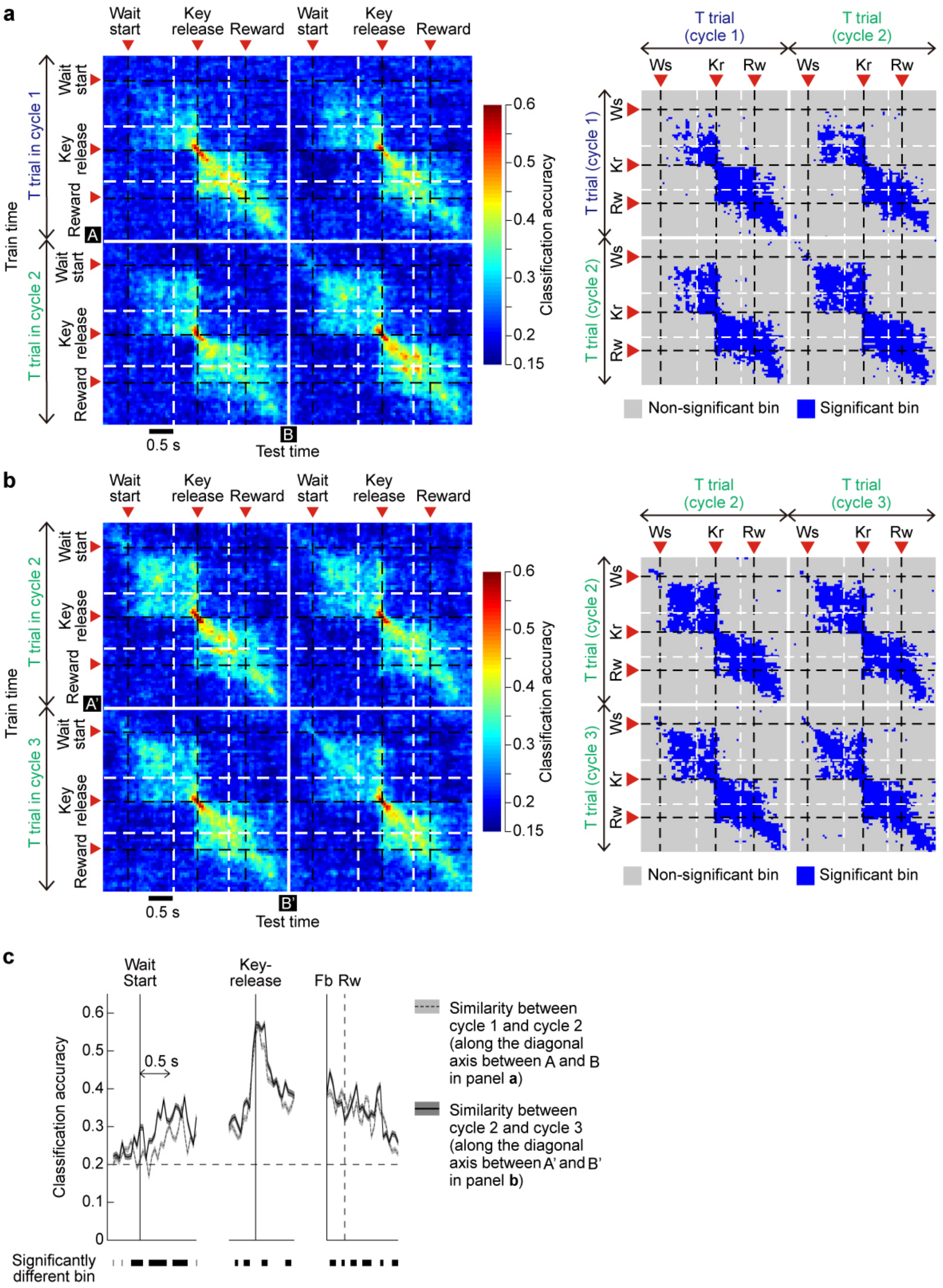
a
Wait start
Key release
Reward
Train time
T trial in cycle 1
T trial in cycle 2
Test time
0.5 s
A
B
Classification accuracy
0.6
0.5
0.4
0.3
0.2
0.15
T trial (cycle 1)
T trial (cycle 2)
Ws
Kr
Rw
Non-significant bin
Significant bin
b
T trial in cycle 2
T trial in cycle 3
A'
B'
T trial (cycle 3)
c
Wait Start
Key-release
Fb Rw
0.5 s
Classification accuracy
Similarity between cycle 1 and cycle 2 (along the diagonal axis between A and B in panel a)
Similarity between cycle 2 and cycle 3 (along the diagonal axis between A' and B' in panel b)
Significantly different bin

Figure S4. Oculomotor sequential memory task: cross-temporal decoding analysis in all periods of T trials across two consecutive cycles. (**a**) Results of cross-temporal decoding analysis across cycles 1 and 2. Conventions as in **Figure S2**. (**b**) Same as in **a**, but for across cycles 2 and 3. (**c**) Difference in the degree of cross-generalization of location codes across cycles between panels **a** and **b**. Black curves indicate classification accuracies along the axis between outlined characters A and B in the color map in **a**. Dashed grey curves indicate classification accuracies along the axis between outlined characters A' and B' in the color map in **b**. Lower horizontal bars indicate time bins with significant difference between the two curves (FDR-controlled permutation test, $p < 0.05$).

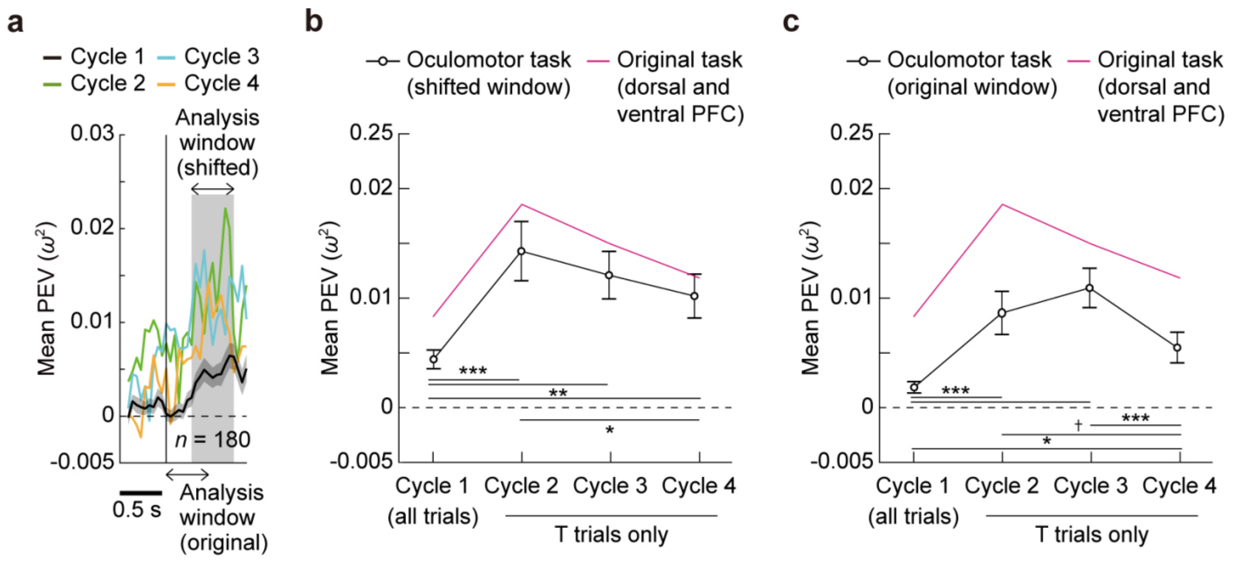

Figure S5. Location selectivity in the oculomotor version of the sequential memory task. (**a**) Time series of PEV in prefrontal cells (n = 180) before and after the wait-start time for each individual cycle in the oculomotor version of the sequential memory task. (**b**) Comparison of mean PEV in the wait period (shaded area in **a**) across cycles (n = 180). In the oculomotor sequential memory task, the analysis time window for the wait period is shifted by 300 ms in order to accommodate delayed peaks of the PEV time-series as compared to the original sequential memory task. Asterisks indicate significantly different comparisons in the *post-hoc* simple effect ANOVA. Purple line indicates the result from the prefrontal population in the original sequential memory task (**Fig. 3d**). (**c**) Same as in **b**, but for the result obtained in the original analysis time window (0 – 500 ms from the wait-start time). Error bars indicate SEM. All other conventions as in **Figure 3**. Source data are provided as a Source Data file.

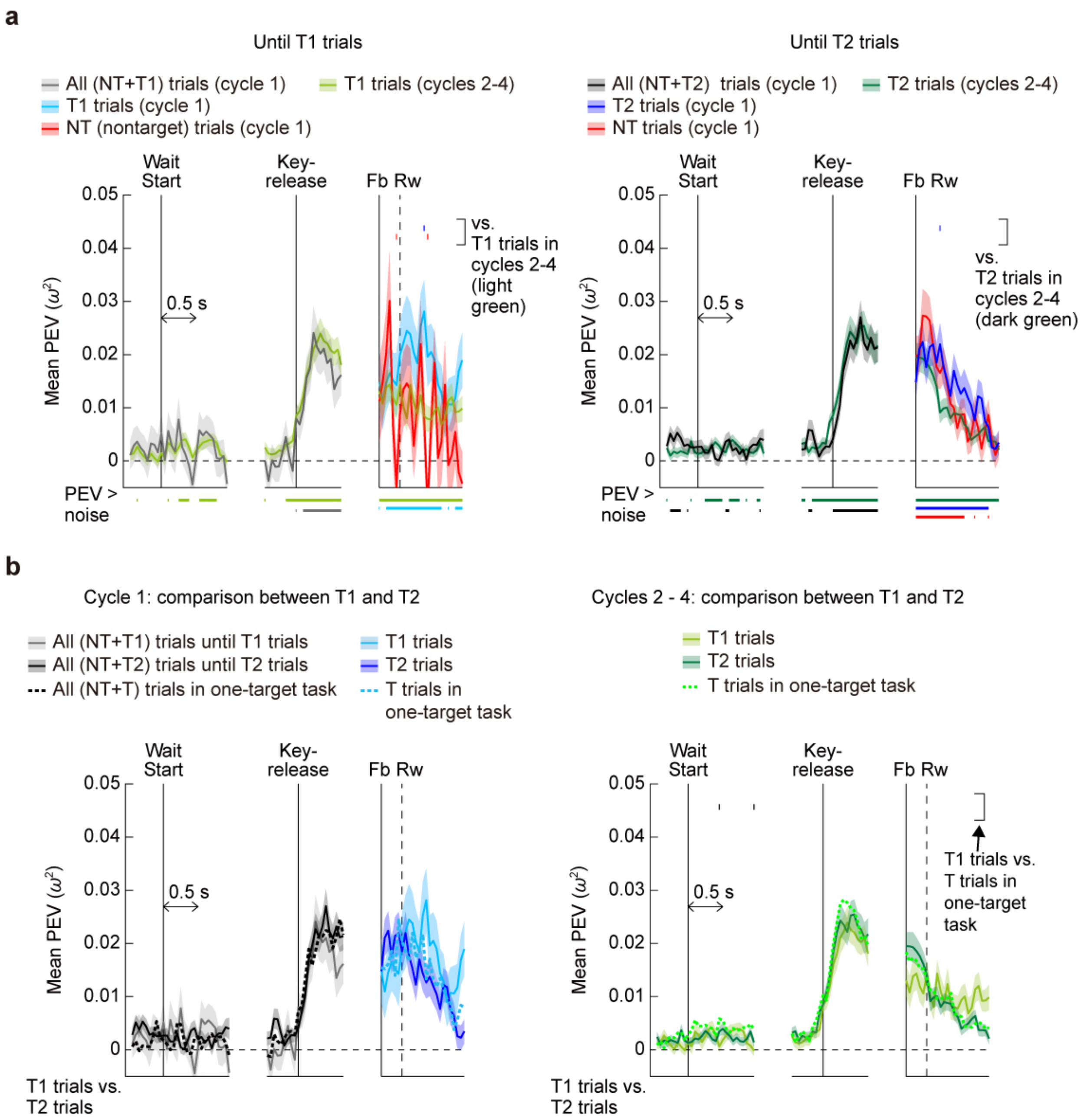

Figure S6. Location selectivity in the superior PPC in the two-target task. (**a**) Time-series of population-averaged PEV (n = 317). Conventions as in **Figure 6a**. (**b**) Comparison of PEV between T1 and T2 trials, separately shown for cycle 1 (left) and cycles 2-4 (right). Conventions as in **Figure 6c**.

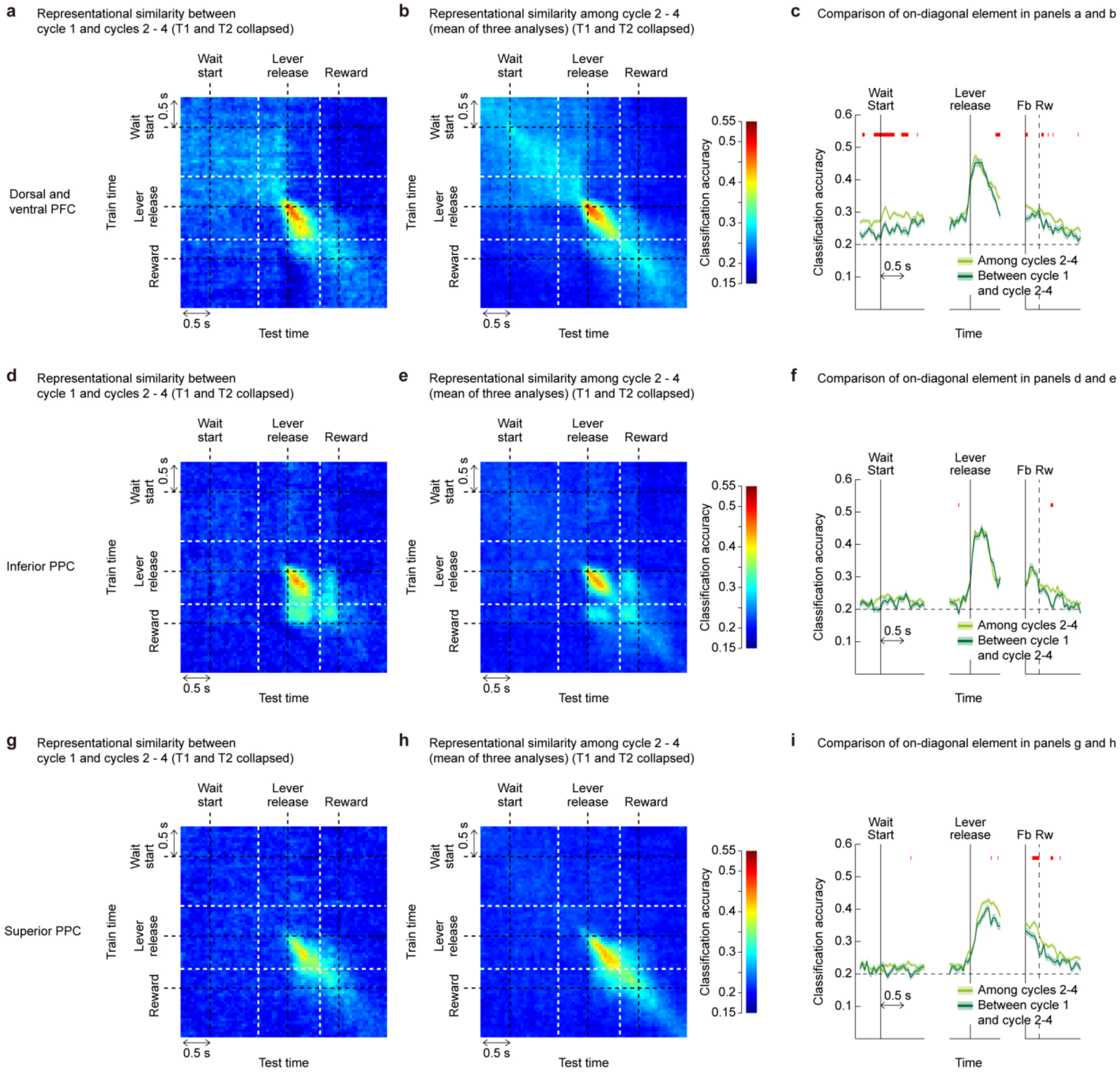

Figure S7. Evidence for processing of target memory during the wait period in the two-target task. (**a**) Dorsal and ventral PFC (n = 498). Between-phase analysis, same as in **Figure7a**. (**b**) Dorsal and ventral PFC. Within-phase analysis, similarity of location coding among cycles 2 – 4. The result illustrates the mean of three within-phase analyses concerning the data in cycles 2 – 4 (see text). (**c**) Dorsal and ventral PFC. Comparison of classification accuracy between panels **a** and **b**. Dark green, on-diagonal elements in **a**. Light green, on-diagonal elements in **b**. Red horizontal bars indicate periods of significant difference between on-diagonal elements in **a** and on-diagonal elements in **b**. (**d-f**) Same as in **a-c**, but for the inferior PPC (n = 252). (**g-i**) Same as in **a-c**, but for the superior PPC (n = 317).

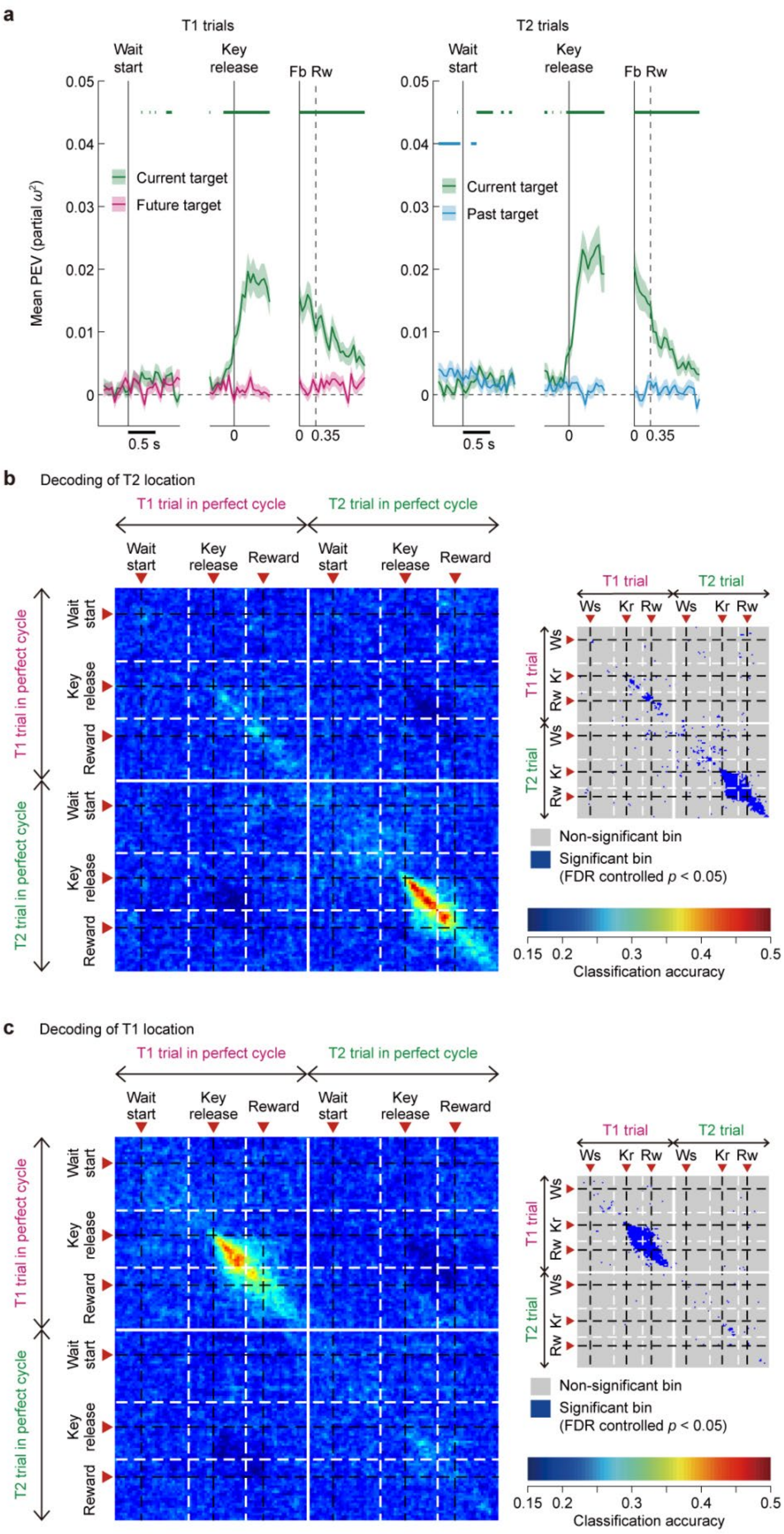

Figure S8. Encoding of past, present and future targets in perfect cycles in superior PPC. (**a**) Time series of PEV for current (green), future (magenta) and past (cyan) targets in T1 (left column) and T2

(right column) trials in perfect cycles (*n* = 317). Conventions as in **Figure 8a**. (**b**) Decoding of T2 target location across successive T1 and T2 trials in perfect cycles. Conventions as in **Figure 8b**. (**c**) Decoding of T1 target location across successive T1 and T2 trials in perfect cycles. Conventions as in **Figure 8c**.

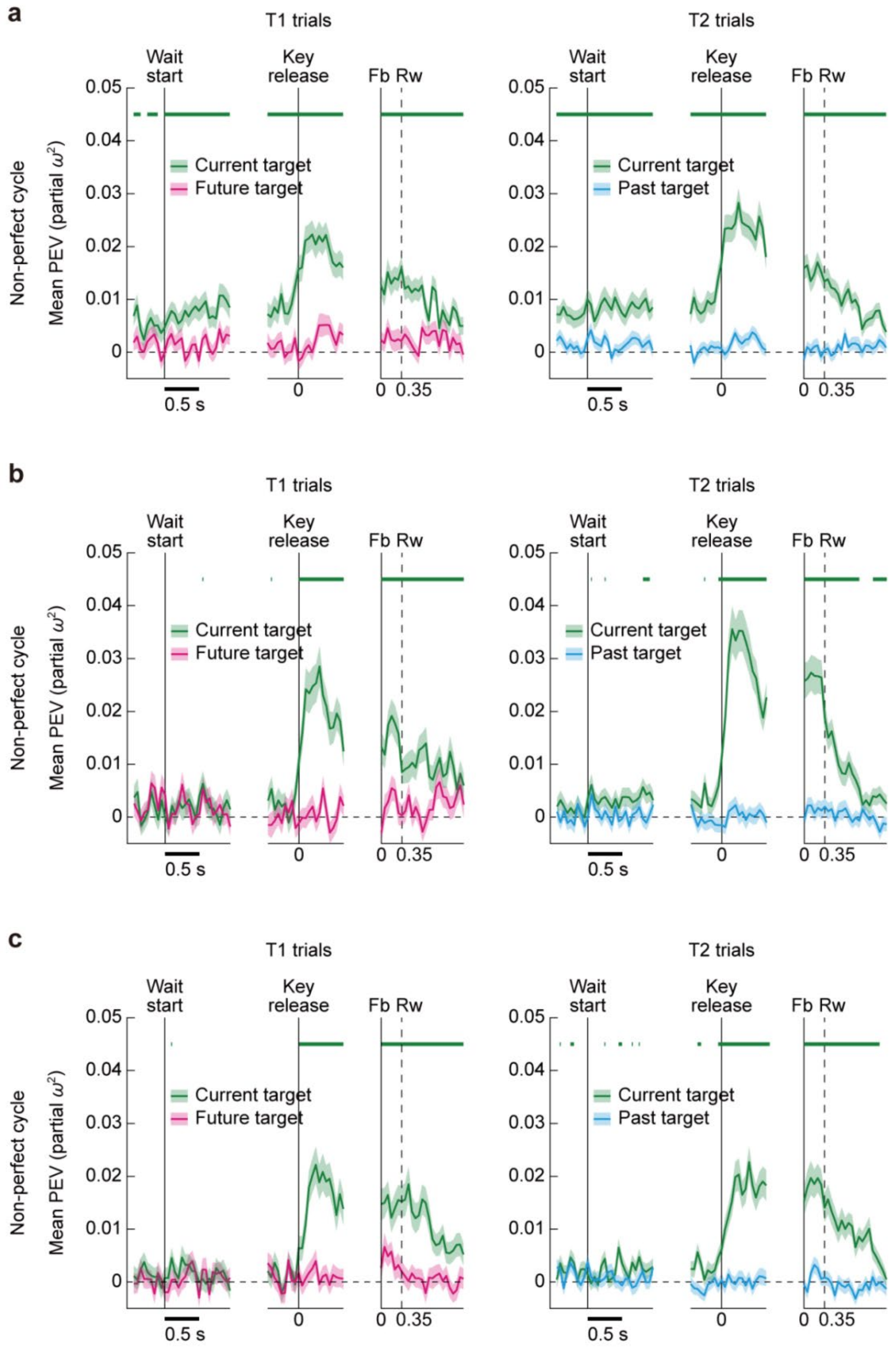

Figure S9. Non-perfect cycles: Encoding of past, present and future targets in two-target problems. (**a**) PFC: Time-series of PEV for current (green), future (magenta) and past (cyan) targets in T1 (left column) and T2 (right column) trials (n = 498). Conventions as in **Figure 8a**. (**b**) Same as in **a**, but for inferior PPC (n = 252). (**c**) Same as in **a**, but for superior PPC (n = 317). Note that in non-perfect cycles, past and future targets corresponded to the non-current targets (i.e., targets selected on non-current trials), but not necessarily the locations actually selected on previous or next trials, unlike in perfect cycles.
