# Supplementary material for: Cycles of goal silencing and reactivation underlie complex problem-solving in primate frontal and parietal cortex
Source: Nat Commun. 2023 Aug 19;14:5054. doi: 10.1038/s41467-023-40676-1 (PMC10439911; doi:10.1038/s41467-023-40676-1)
Supplement: Supplementary file 2 — Reporting Summary [file 41467_2023_40676_MOESM2_ESM.pdf]

nature portfolio

Corresponding author(s): Kei Watanabe

Last updated by author(s): 2023/06/30

Reporting Summary

Nature Portfolio wishes to improve the reproducibility of the work that we publish. This form provides structure for consistency and transparency in reporting. For further information on Nature Portfolio policies, see our Editorial Policies and the Editorial Policy Checklist.

Statistics

For all statistical analyses, confirm that the following items are present in the figure legend, table legend, main text, or Methods section.

n/a	Confirmed	
☐	☒	The exact sample size (*n*) for each experimental group/condition, given as a discrete number and unit of measurement
☐	☒	A statement on whether measurements were taken from distinct samples or whether the same sample was measured repeatedly
☐	☒	The statistical test(s) used AND whether they are one- or two-sided<br>*Only common tests should be described solely by name; describe more complex techniques in the Methods section.*
☐	☒	A description of all covariates tested
☐	☒	A description of any assumptions or corrections, such as tests of normality and adjustment for multiple comparisons
☐	☒	A full description of the statistical parameters including central tendency (e.g. means) or other basic estimates (e.g. regression coefficient) AND variation (e.g. standard deviation) or associated estimates of uncertainty (e.g. confidence intervals)
☐	☒	For null hypothesis testing, the test statistic (e.g. F, t, r) with confidence intervals, effect sizes, degrees of freedom and P value noted<br>*Give P values as exact values whenever suitable.*
☒	☐	For Bayesian analysis, information on the choice of priors and Markov chain Monte Carlo settings
☒	☐	For hierarchical and complex designs, identification of the appropriate level for tests and full reporting of outcomes
☐	☒	Estimates of effect sizes (e.g. Cohen's *d*, Pearson's *r*), indicating how they were calculated

Our web collection on statistics for biologists contains articles on many of the points above.

Software and code

Policy information about availability of computer code

Data collection	Task event timing, monkey's behavioral response and eye movement data were collected by a REX system (monkeys A and B) and a TEMPO system (monkey C). For analysis of spiking activity, the raw neural signal was filtered (300 Hz to 10 kHz) by Cerebus System (Blackrock Microsystems) in monkeys A and B, and by RZ2Bioamp Processor (Tucker-Davis Technologies, Fl) in monkey C. Spikes were sorted using Plexon Offline Sorter version 2.8.8. Matlab was used for creating experimental data files.
Data analysis	Data analysis was carried out in MATLAB (2018b). Decoding analysis was conducted using the MATLAB Neural Decoding Toolbox (Meyers, E. 2013).<br>The codes to generate the main figures and results are openly available in the G-node GIN repository at: https://gin.g-node.org/KeiWatanabe/Watanabe_Kadohisa_Kusunoki_Buckley_Duncan.git

For manuscripts utilizing custom algorithms or software that are central to the research but not yet described in published literature, software must be made available to editors and reviewers. We strongly encourage code deposition in a community repository (e.g. GitHub). See the Nature Portfolio guidelines for submitting code & software for further information.

Data

Policy information about availability of data

All manuscripts must include a data availability statement. This statement should provide the following information, where applicable:

- Accession codes, unique identifiers, or web links for publicly available datasets
- A description of any restrictions on data availability
- For clinical datasets or third party data, please ensure that the statement adheres to our policy

The raw data that support the findings of this study are openly available in the G-node GIN repository at: https://gin.g-node.org/KeiWatanabe/Watanabe_Kadohisa_Kusunoki_Buckley_Duncan.git

Research involving human participants, their data, or biological material

Policy information about studies with human participants or human data. See also policy information about sex, gender (identity/presentation), and sexual orientation and race, ethnicity and racism.

Reporting on sex and gender	Not applicable.
Reporting on race, ethnicity, or other socially relevant groupings	Not applicable.
Population characteristics	Not applicable.
Recruitment	Not applicable.
Ethics oversight	Not applicable.

Note that full information on the approval of the study protocol must also be provided in the manuscript.

Field-specific reporting

Please select the one below that is the best fit for your research. If you are not sure, read the appropriate sections before making your selection.

☒ Life sciences ☐ Behavioural & social sciences ☐ Ecological, evolutionary & environmental sciences

For a reference copy of the document with all sections, see nature.com/documents/nr-reporting-summary-flat.pdf

Life sciences study design

All studies must disclose on these points even when the disclosure is negative.

Sample size	The sample sizes in the present study are similar to those typically used in the field (e.g., Panichello and Buschman, 2021; Katsuki and Constantinidis, 2012). No statistical methods were used to determine sample size.
Data exclusions	We did not exclude any animal or neurons from data analysis.
Replication	Our results in behavioral analysis were replicated in all of the three animals used in this study. The main part of the neural results (i.e., observations concerning memory reactivation) was replicated in two different groups of animals (monkeys A, B and monkey C) each performing one of the two variants of the task used in this study.
Randomization	All animals were used for experiments. Trial conditions were randomly determined by a computer program.
Blinding	Data analysis was not performed blind to the conditions of the experiments. Because the same set of analysis was performed across experimental conditions, blinding is not relevant for this study. In addition, during experiments, the blocks of different trial types were randomly ordered (alternated) by a computer program.

Reporting for specific materials, systems and methods

We require information from authors about some types of materials, experimental systems and methods used in many studies. Here, indicate whether each material, system or method listed is relevant to your study. If you are not sure if a list item applies to your research, read the appropriate section before selecting a response.

Materials & experimental systems

n/a	Involved in the study
☒	☐ Antibodies
☒	☐ Eukaryotic cell lines
☒	☐ Palaeontology and archaeology
☐	☒ Animals and other organisms
☒	☐ Clinical data
☒	☐ Dual use research of concern
☒	☐ Plants

Methods

n/a	Involved in the study
☒	☐ ChIP-seq
☒	☐ Flow cytometry
☐	☒ MRI-based neuroimaging

Animals and other research organisms

Policy information about studies involving animals; ARRIVE guidelines recommended for reporting animal research, and Sex and Gender in Research

Laboratory animals	We used two male Macaca mulatta (Rhesus monkey, monkeys A and B, each 13 kg, eight years old) and one female Macaca fuscata (Japanese monkey, monkey C, 8 kg, nine years old).
Wild animals	This study did not involve wild animals.
Reporting on sex	We used both male and female subjects. Comparable results were obtained for animals of both sexes, as reported in the manuscript (e.g., compare Figures 3 vs 5, and Figures 4 and S4) ; .
Field-collected samples	This study did not involve field-collected samples.
Ethics oversight	The experiments were conducted in accordance with the Animals (Scientific Procedures) Act 1986 of the UK; all procedures were licensed by a Home Office Project License obtained after reviewed by Oxford University's Animal Care and Ethical Review committee. The experiments concerning monkey C were also approved by the Animal Research Committee at the Graduate School of Frontier Biosciences, Osaka University and were in full compliance with the guidelines of the National BioResource Project "Japanese Macaques".

Note that full information on the approval of the study protocol must also be provided in the manuscript.

Magnetic resonance imaging

Experimental design

Design type	Anesthetized structural scan
Design specifications	Not applicable.
Behavioral performance measures	Not applicable.

Acquisition

Imaging type(s)	Structural
Field strength	3.0 Tesla
Sequence & imaging parameters	(1) 3D Gradient echo, MP-R AGE , FOV = 64mm, matrix size= 128 x 128, number of slices= 128, voxel size= 0.5 mm (isotropic), TR=1500ms/TE=3 .29ms/FA=9deg<br>(2) 2D Spin echo, FOV = 144mm, matrix size= 192 x 192, number of slices= 80, voxel size= 0.75 x 0.75 x 0.8mm, TR=12580ms/TE=67ms/FA=160deg
Area of acquisition	Whole brain

Diffusion MRI ☐ Used ☒ Not used

Preprocessing

Preprocessing software	SPM12
Normalization	Not applicable.
Normalization template	Not applicable.

Noise and artifact removal | Not applicable.

Volume censoring | Not applicable.

Statistical modeling & inference

Model type and settings | Not applicable.

Effect(s) tested | Not applicable.

Specify type of analysis: ☒ Whole brain ☐ ROI-based ☐ Both

Statistic type for inference (See Eklund et al. 2016) | Not applicable.

Correction | Not applicable.

Models & analysis

n/a	Involved in the study
☒	☐ Functional and/or effective connectivity
☒	☐ Graph analysis
☒	☐ Multivariate modeling or predictive analysis
